# microGalaxy: A gateway to tools, workflows, and training for reproducible and FAIR analysis of microbial data

**DOI:** 10.1101/2024.12.23.629682

**Published:** 2024-12-27

**Authors:** Engy Nasr, Pierre Amato, Anshu Bhardwaj, Daniel Blankenberg, Daniela Brites, Fabio Cumbo, Katherine Do, Emanuele Ferrari, Timothy J. Griffin, Björn Grüning, Saskia Hiltemann, Pratik Jagtap, Subina Mehta, Kimberly Métris, Saim Momin, Asime Oba, Christina Pavloudi, Nikos Pechlivanis, Raphaëlle Péguilhan, Fotis Psomopoulos, Nedeljka Rosic, Michael C. Schatz, Valerie Claudia Schiml, Cléa Siguret, Nicola Soranzo, Andrew Stubbs, Peter van Heusden, Mustafa Vohra, microGalaxy Community, Paul Zierep, Bérénice Batut

**Affiliations:** 1 Bioinformatics Group, Department of Computer Science, University of Freiburg, Georges-Koehler-Allee 106, D-79110 Freiburg, Germany; 2 Université Clermont Auvergne, CNRS, INP Clermont Auvergne, Institut de Chimie de Clermont-Ferrand (ICCF), F-63000, Clermont-Ferrand, France; 3 Université Clermont Auvergne, CNRS, Laboratoire Microorganismes: Génome et Environnement, Clermont-Ferrand, France; 4 CSIR-Institute of Microbial Technology, Chandigarh, and Academy of Scientific & Innovative Research (AcSIR), Delhi, India; 5 Center for Computational Life Sciences, Cleveland Clinic, Cleveland, Ohio, USA; 6 Swiss Tropical and Public Health Institute, University of Basel, Basel, Switzerland; 7 University of Minnesota, Minneapolis, MN, USA; 8 National Research Council of Italy – Water Research Institute (CNR-IRSA) Molecular Ecology Group (MEG), Verbania, Italy; 9 University of Freiburg, Faculty of Chemistry and Pharmacy, Freiburg, Germany; 10 Department of Genetics and Biochemistry, Clemson University, Clemson, SC, USA; 11 University of Maiduguri, Maiduguri, Nigeria; 12 European Marine Biological Resource Centre (EMBRC-ERIC), Paris, France; 13 Institute of Applied Biosciences, Centre for Research and Technology Hellas, Thermi, 57001, Thessaloniki, Greece; 14 Department of Chemical and Biochemical Engineering, Technical University of Denmark, DK-2800 Kgs. Lyngby, Denmark; 15 Faculty of Health, Southern Cross University, Gold Coast, QLD, Australia; 16 Department of Computer Science, Johns Hopkins University, Baltimore, Maryland 21218, USA; 17 The Protein Engineering and Proteomics Group (PEP), Faculty of Chemistry, Biotechnology and Food Science, Norwegian University of Life Sciences, Elizabeth Stephansens vei 15, 1433 Ås, Norway; 18 IFB-core, Institut Français de Bioinformatique, CNRS, INSERM, INRAE, CEA, Evry, France; 19 Plateforme AuBi, Mésocentre Clermont-Auvergne, Université Clermont Auvergne, 7 avenue Blaise Pascal, 63170 Aubière, France; 20 Earlham Institute, Norwich, UK; 21 Department of Pathology and Clinical Bioinformatics, Erasmus MC Cancer Institute, Erasmus MC, Rotterdam, Netherlands; 22 South African Medical Research Council Bioinformatics Unit, South African National Bioinformatics Institute, University of the Western Cape; 23 Department of Medical Laboratory Science, Lovely Professional University, Punjab 144411, India; 24 Department of Microbiology, Shri Vinoba Bhave Civil Hospital, Silvassa 396230, India

## Abstract

Microbial research generates vast and complex data from diverse omics technologies, necessitating innovative analytical solutions. microGalaxy (Galaxy for Microbiology) addresses these needs with a user-friendly platform that integrates >220 tool suites and >65 curated workflows for microbial analyses, including taxonomic profiling, assembly, annotation, and functional analysis. Hosted on the main EU Galaxy server (microgalaxy.usegalaxy.eu), it supports workflow creation & customization, sharing, and updates across public and private Galaxy servers, ensuring flexibility and reproducibility. The platform also offers 45+ tutorials, 15+ instructional videos, and structured learning pathways, empowering researchers to conduct advanced analyses. Backed by a community-driven approach, microGalaxy prioritizes tool testing, semi-automatic updates, and multi-omics integration to meet global research demands. With its focus on rapid workflow prototyping and high-throughput processing, microGalaxy provides scalable resources for researchers at all expertise levels, enabling them to tackle challenges in microbial data analysis with confidence and efficiency.

## Graphical Abstract

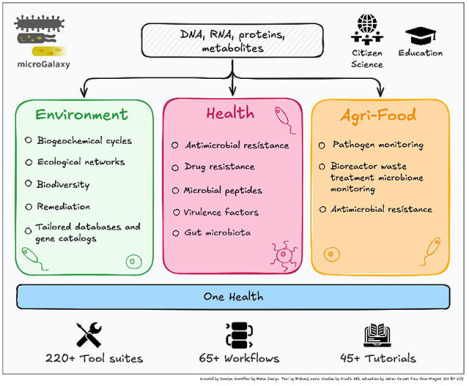

## Main text

### Introduction

Microbiology has experienced a transformative shift over the past two decades with advances in molecular biology and high-throughput sequencing technologies^1,2^. These innovations have greatly enhanced our ability to characterise the composition and functional properties of microorganisms in diverse clinical, environmental, and agricultural contexts. However, the massive volume and diversity of generated data presents substantial challenges, particularly in processing, interpreting, and deriving meaningful insights. Effectively addressing these challenges requires bioinformatics solutions that are not only robust, accurate and reproducible but also follow FAIR (Findable, Accessible, Interoperable, Reusable)^3^ principles.

Microbiology research faces several key challenges. The vast scale of microbiological data, generated by diverse methods such as short-read and long-read sequencing and mass spectrometry, makes analysis and processing complex. Integrating data from multiple sources and experimental designs, including (meta)genomics, (meta)transcriptomics, and (meta)proteomics, demands extensive expertise, robust computational resources, and access to comprehensive databases. Choosing appropriate bioinformatics tools, parameters, and reference databases further adds to the complexity and risks introducing biases. Moreover, access to computational infrastructure is globally uneven, limiting researchers’ ability to perform large-scale data analysis. These disparities highlight the urgent need for an open, standardized, and freely accessible computing environment for microbiological data analysis.

The growing emphasis on FAIR^3^ principles and open practices presents an opportunity for researchers to adopt more transparent and reproducible workflows, but adds another layer of complexity. The challenges include focused efforts in organizing, annotating, and curating data, as well as ensuring compatibility between diverse tools and platforms. Nevertheless, these approaches are particularly valuable in interdisciplinary fields such as One Health^4^, where microbiology intersects with public health, agriculture, and environmental science, fostering collaboration and innovation across domains.

To address these needs, several platforms and systems have been developed including MGnify^5^, MG-RAST^6^, bioBakery^7^, Seqera Tower, Epi2Me Desktop, SILVAngs, Anvi’o^8^, KBase^9^, BV-BRC^10^, and iMetaLab^11^ (Supplementary Table 1). These platforms excel in specific domains such as genomics, transcriptomics, or proteomics, offering targeted solutions for particular types of analysis. However, many are limited to specific ‘omics modalities or analysis workflows and may lack interoperability or comprehensive documentation. They may also work as a “black-box”, thus reducing user transparency and control over key analytical steps. While these platforms are invaluable for their intended purposes, they often do not answer the broader need for an open, integrated, flexible solution that spans diverse microbial data types and analytical requirements. They must also remain accessible to researchers with limited computational expertise.

In this work, we present microGalaxy community efforts to build an open-source Galaxy-based platform designed to provide a gateway for microbial data analysis. Galaxy is an open, web-based platform for data-intensive computational research, built on FAIR principles to ensure accessibility, reproducibility, and interoperability of data analysis^12^. Galaxy has been widely adopted by the scientific community, with 12,545 citations in scientific papers across just 8 of the major Galaxy papers and a consistent upward trend (Supplementary Figure 1A); of these, 26.65% are associated with microbiological data. Bacteria and microbiomes remain the most frequently studied objects, with functional analysis and isolate-based studies being key technical targets (Supplementary Figure 1B). Among methodologies, metagenomics and related approaches dominate (Supplementary Figure 1B).

A community survey conducted in 2023 highlighted the platform’s significant impact and identified key areas for growth (Supplementary Document 1). Coherent with Galaxy citations (Supplementary Figure 1A), the survey revealed that bacteria are a primary research focus on Galaxy, with metagenomics and single-organism genomics being the most widely used techniques, involving functional analysis, gene identification, and assembly analysis, which are computationally intensive (Supplementary Figure 2, Supplementary Table 2). The respondents also pointed out two major obstacles preventing them from effectively analysing their data with Galaxy: a lack of experience and technical difficulties. These challenges are being actively addressed through ongoing Galaxy updates and expanding training resources. Moreover, the survey emphasised a notable underrepresentation of researchers from the Global South, a group microGalaxy aims to support through its free computational resources. Notably, most survey participants indicated that they would adopt microbial tools if these were available on Galaxy. This survey underscores the essential role of the microGalaxy community in improving access to microbial tools and expanding their reach to a more diverse global audience.

microGalaxy builds on Galaxy’s foundation to address the challenges of microbial bioinformatics by offering: (i) access to a wide variety of critically reviewed and evaluated software tools and workflows covering diverse analysis types, (ii) a graphical interface and workflow editor that simplifies complex analyses, (iii) free access to public computational resources in standardised environments, leveling the field for researchers worldwide, (iv) integration with training resources to empower users with varying levels of bioinformatics expertise, (v) a powerful Application Programming Interface (API) that enables automation, integration, and advanced data analysis. By leveraging these unique features, microGalaxy provides a framework that supports the global microbiology community in overcoming data and computational challenges and advancing the field.

### Results

#### A microbial research focused Galaxy Lab for reproducible and FAIR data analysis

The microGalaxy platform is built using the Galaxy framework^12^. Galaxy provides a user-friendly interface, allowing users to perform data analyses without requiring programming expertise. Its functionality enables analytical tools to be used individually or combined into complex workflows. Galaxy also implements provenance tracking, documenting every analysis step and workflow invocation, which enable users to replicate and build on others’ research.

The microGalaxy Lab leverages Galaxy to provide a customised view with a dedicated collection of tools, workflows, and resources specifically tailored to microbial research. It is available on the European Galaxy server (microgalaxy.usegalaxy.eu), thus providing free access to individual storage and computational resources up to server-specific limits, allowing worldwide users, lacking local computational infrastructure, to conduct sophisticated analyses.

#### Community-curated tools for microbial research

**Fig 1.**
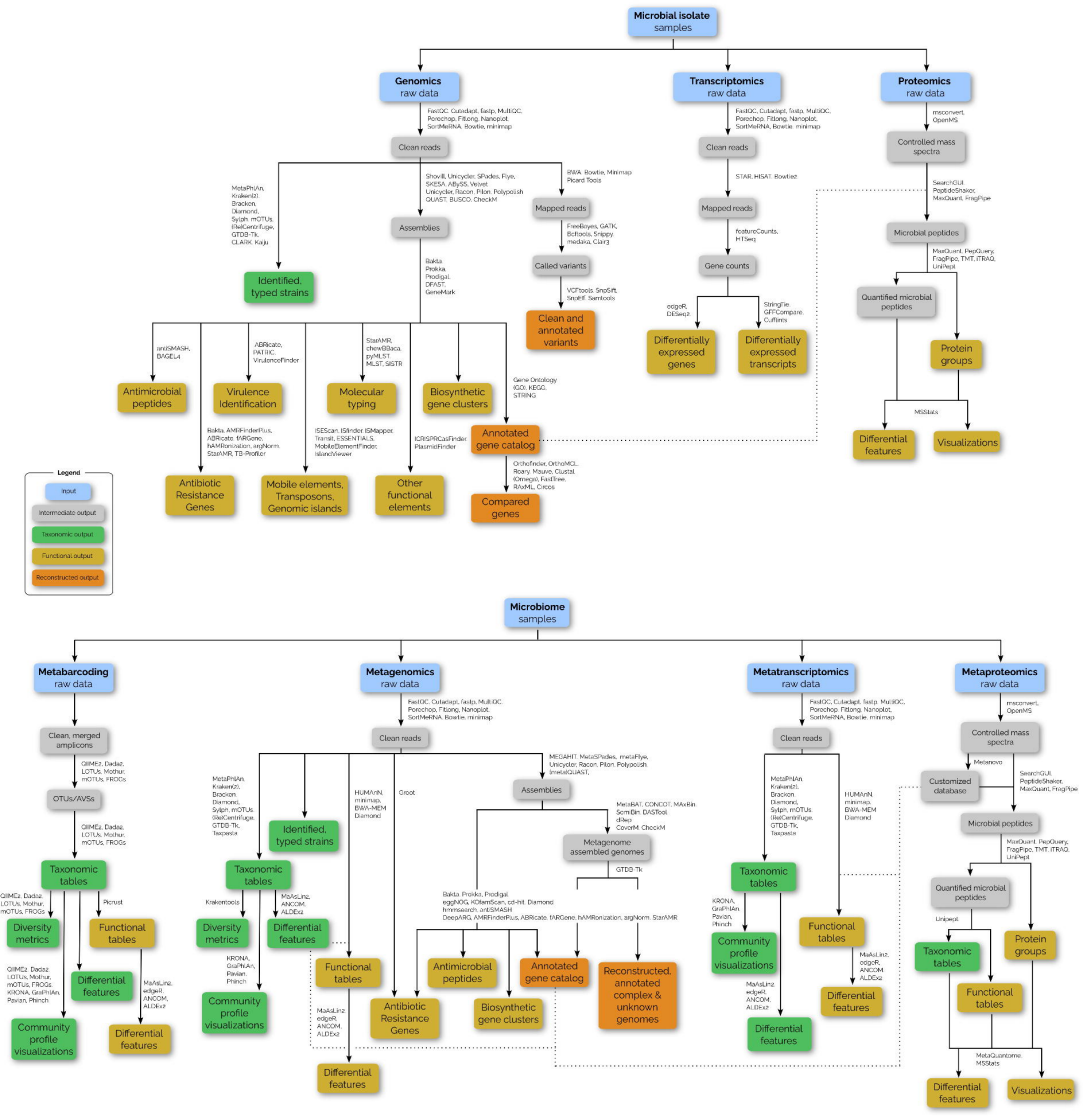
Overview of microbial data analysis tasks with corresponding tool suites and potential workflows available on microGalaxy.

Of the over 10,000 scientific tools^12^ available within the Galaxy ecosystem in 1,500 tool suites, microGalaxy provides a refined set of 224 tool suites specifically curated for microbial research (Figure 1, Supplementary Table 3), steadily growing over time (Supplementary Figure 3C) and well-suited for routine and real-world research practice. Collectively, these tools have been used more than 17 Million times across all major Galaxy servers over the past five years, highlighting their wide adoption (Supplementary Figure 3, Supplementary Table 3).

This figure illustrates the diverse range of microbial analysis tasks, grouped by sample (e.g., microbial isolates and microbiomes) and data types (e.g., genomics, transcriptomics, proteomics, and multi-omics approaches). Each task is linked to intermediate and final outputs, with annotations indicating the tool suites that could be used for these analyses.

Users have several ways to access data within microGalaxy. In addition to local uploads and bulk transfer via HTTP or the commercial clouds, a variety of tools are available to automatically fetch data from multiple sources, including UCSC Genome Browser database^13^, NCBI Sequence Read Archive (SRA)^14^, EMBL-EBI European Nucleotide Archive (ENA)^15^ and MGnify^5^. Relatedly, Galaxy hosts a large collection of reference databases, essential for tasks such as functional annotations and taxonomic classification. For example, usegalaxy.eu offers over 170 integrated reference genomes and overall 11 Terabyte of reference data. These data are shared using CernVM-FS^16^, which is a network file system optimized for delivering and distributing scientific software and datasets to computing resources in large-scale distributed computing environments

The set of curated tool suites in microGalaxy supports a comprehensive range of bioinformatics operations, such as genome assembly, binning, taxonomic classification, variant calling, and protein quantitation, and spans diverse scientific topics including metagenomics, phylogenetics, microbial ecology, and metaproteomics (Figure 1, Supplementary Figure 3B–C). The collected usage statistics reflect the alignment of microGalaxy with the needs of the microbial research community for sequence pre-processing (e.g. fastp^17^, Cutadapt^18^), Antimicrobial resistance prediction (e.g. ABRicate), genome annotation (e.g. Prokka^19^), genome assembly (e.g. (meta)SPAdes^20^) and taxonomic classification (e.g. mothur^21^, Kraken2^22^) tools. In contrast, more specialised tools for areas such as microbial ecology and phylogenetics see focused application within their respective fields, catering to advanced and emerging analyses (Supplementary Figure 3B–C). This balance ensures that microGalaxy accommodates both routine, as well as specialised, data analysis needs, allowing researchers to explore diverse microbial research or surveillance questions efficiently.

Another standout feature of Galaxy is its support for interactive tools, which provide fully functional web applications directly within the Galaxy platform. These tools can utilize Galaxy-generated data to perform complex tasks requiring live user interaction. In addition to general-purpose tools like Jupyter Notebooks and RStudio instances, Galaxy offers a collection of specialised interactive tools specifically for microbial research, including Pavian^23^, Phinch^24^, Shiny Phyloseq^25^, and Apollo^26^.

The tools are not only available within the microGalaxy platform, but also on various public Galaxy instances, including specialised instances such as GalaxyTrakr^27^ and Galaxy@Pasteur (Supplementary Figure 3A). The widespread availability of core microbial analysis tools across these servers guarantees that researchers can rely on consistent and reliable resources for their workflows, regardless of the server they choose.

#### Integrated Workflows for Scalable Microbial Analyses

Workflows are central to data analysis demands that require the need to connect multiple tools in a reproducible manner. Galaxy offers a robust workflow management system comparable to Nextflow^28^ and Snakemake^29^, but with the essential advantage of a graphical interface. Users can build complex analytical pipelines by connecting tools with intuitive ‘noodles’ on the workflow canvas or extracting workflows from manually performed analysis.

microGalaxy provides a comprehensive collection of 69 community-curated, ready-to-use, well-documented workflows tailored to microbial research (Supplementary Table 4, Supplementary Figure 4). These workflows can be executed directly on the microGalaxy platform or installed on any Galaxy server. Twenty of these workflows have been published on workflow repositories such as WorkflowHub^30^ and Dockstore^31^, further extending their accessibility to the broader research community. The other workflows are publicly shared across Galaxy servers with the *microgalaxy* tag, and can be accessed and executed directly on the server where they are found or transferred to other servers, provided the required tools are installed.

Several specialised workflows have been created to facilitate the analysis of microbial data. For instance, ABRomics (https://www.abromics.fr/) – French platform for antibiotic resistance research and surveillance – uses microGalaxy workflows for several essential processes. ABRomics workflows run quality assessments and read cleaning, taxonomic assignment, and assembly of bacterial paired-end short and/or long read data, accompanied by comprehensive quality metrics and reports. After assembly, they include genome annotation steps to identify genes, plasmids, integrons, insertion sequence (IS) elements, and antimicrobial resistance genes, thoroughly examining microbial genomes.

Similarly, PathoGFAIR^32^ workflows enable the detection and tracking of pathogens from metagenomic Nanopore, i.e. long read, sequencing data. PathoGFAIR workflows include interconnected analyses: quality control and contamination filtering, taxonomic profiling, phylogenetic identification, antibiotic resistance gene detection, variant calling, and comprehensive reporting and visualisation. Together, they enhance the reproducibility and accessibility of microbial pathogenesis research.

Beyond genomics, specialised workflows for metaproteomics analysis of microbial communities have been developed. These workflows integrate several tool suites to address all steps in metaproteomics analysis, from database construction to functional and taxonomic analysis. For example, the combination of SearchGUI^33^(RRID:SCR_012054) and PeptideShaker^34^(RRID:SCR_002520) allows multiple search algorithms to be used. After peptide identification, taxonomic data can be assigned using Unipept^35^(RRID:SCR_024987), and functional analysis can be performed through the Gene Ontology database^36^.

microGalaxy also supports multi-omics integration workflows, enabling comprehensive analyses of microbial communities. By combining tools for metagenomic assembly, binning, taxonomic classification, and functional annotations with quantitative metatranscriptomics and metaproteomics data, a set of workflows^37^ facilitates the functional analysis of individual species within a complex microbial ecosystem. In addition, the Galaxy ecosystem is home to a diverse set of software and training material for metabolomics analysis^38–40^ which can be applied to single organism studies or more complex microbiome-regulated systems. These tools focus on both mass spectrometry and NMR generated data and complement the microbiome-focused multi-omic software described in this review.

microGalaxy workflows can also enhance the value of existing pipelines established by other resources. In collaboration with EMBL-EBI, the MGnify^5^ pipeline for amplicon analysis has been imported into a Galaxy workflow, transforming it into a community-modifiable and customizable version of its EMBL-EBI counterpart.

#### Resources Backed by Comprehensive Training Support

Training empowers users to use complex analytical processes, deepen their understanding of the methodologies, and enhance data interpretation skills. Comprehensive training support for tools and workflows ensures that researchers of all experience levels can fully utilise microGalaxy resources (Supplementary Figure 5). A total of 48 tutorials, 17 videos (16 hours), and 4 structured learning pathways for microbial analyses are currently hosted on the Galaxy Training Network (GTN)^41,42^. These tutorials cover various topics and operations, from basic sequence analysis to advanced areas like metaproteomics or metagenomics assembly (Supplementary Figure 5). Regular updates to these resources ensure up-to-dateness with the latest tools and methodologies. Integrating these materials into microGalaxy directly enhances accessibility, empowering users to confidently undertake complex analyses.

Beyond self-guided resources, training events are critical in building skills and fostering confidence in using Galaxy, tools, and workflows. Over the last five years, the microGalaxy community has conducted more than 30 training events (Supplementary Table 5). Many of these events are supported by the Galaxy Training Infrastructure as a Service (TIaaS) framework^43^, which provides dedicated computing resources to facilitate fast and efficient data processing for training sessions. A notable example is the “Analysis of Functions Expressed by Microbiomes (https://galaxyproject.org/events/2021-11-microbiomes/home/)” workshop, held in November 2021 and co-hosted by CSIR-IMTech (Chandigarh, India) and Galaxy-P team (MN, USA). This workshop taught 37 participants with hands-on experience in Galaxy tools for analysing microbiome functions, including metagenomic and metatranscriptomic data, while encouraging collaboration and knowledge sharing among researchers exploring the ecological roles of microbial communities.

The microGalaxy community also contributes significantly to large-scale global initiatives like the Galaxy Training Academy (previously Galaxy Smörgåsbord). Launched in 2021, this event has run annually, attracting over 10,000 registrations. These fully remote, highly flexible, asynchronous, video-based programs offer support through the Galaxy community on Slack. Participants can design personalised, self-paced learning experiences tailored to their interests and expertise while accommodating their unique schedules. During the 2022 and 2023 events, metagenomics tutorials accounted for 5.36% and 7.76% of all completed tutorials. In 2024, the microGalaxy community introduced two dedicated tracks: Microbiome and Bacterial Genomics, with 66.69% of over 3,000 registrants expressing an interest in at least one of these tracks. The same approach has been used to deliver several trainings (e.g. https://training.galaxyproject.org/training-material/events/2024-06-10-mtb-ngs.html) in genomic analysis of *Mycobacterium tuberculosis*, reaching a broad participation from countries in the global south, where tuberculosis is one of the most important causes of mortality and morbidity due to infection.

#### Robust Support and Community Engagement

In addition to comprehensive training resources, microGalaxy users benefit from robust support mechanisms; assistance is readily available. The microGalaxy community plays an essential role through the Galaxy Help forum (https://help.galaxyproject.org/) and community-driven platforms like Matrix (https://matrix.to/#/#galaxyproject_microGalaxy:gitter.im) and Slack, ensuring that users can get help when needed. This active and engaged group, includes over 50 members on the mailing list and more than 60 participants in the Matrix chat, fostering a collaborative atmosphere. Regular meetings, working groups, and community-driven initiatives ensure the continuous development and enhancement of microGalaxy. Collaboration events (e.g., hackathons, workshops, regular meetings) are instrumental in bringing members together to collaborate, develop new workflows, improve existing tools, and share expertise. Notably, two dedicated hackathons^44,45^ were conducted with the aim of improving the annotation of microGalaxy tools using the EDAM Ontology (see also Methods section). Furthermore, a recent hackathon on integrating workflows into the Intergalactic Workflow Commission (IWC) (https://github.com/galaxyproject/iwc) has substantially expanded the platform’s capabilities. Other contributors further enrich the microGalaxy resources by contributing novel tools and resources, ensuring that the platform remains state-of-the-art in microbial data analysis.

### Discussion

microGalaxy offers a comprehensive platform that integrates 220+ tools, 65+ workflows, public database access as well as locally installed reference databases, and 45+ training materials for the microbial research fields within the Galaxy ecosystem. It provides a competitive and accessible solution with extensive capabilities in microbial data analysis. microGalaxy is characterized by its open-source nature, workflow management, and integration of diverse analytical tools (Supplementary Table 1). Its user-friendly design and powerful computational resources make it a valuable resource for researchers, enabling them to perform complex analyses that cannot be performed on personal hardware.

#### Exemplary use cases empowered by microGalaxy resources

microGalaxy’s comprehensive resources have been successfully applied to tackle diverse challenges in microbial research, as evidenced by numerous citations (Supplementary Figure 1). Here, we highlight a selection of outstanding research applications, with additional use cases detailed in Supplementary Table 5.

Merdan et al. 2022^46^ used Galaxy to analyse genomic data to identify drug-resistance mutations in *Candida glabrata* fungi. They discovered that the 941delC mutation in the *ERG1* gene, which disrupts the ergosterol synthesis pathway, enables the fungus to utilise exogenous cholesterol, resulting in resistance to azole and AmB antifungal drugs.

Applying machine learning (ML) and artificial intelligence (AI) to microbiome research is an area of growing interest. Incorporating AI-driven tools like chopin2 into Galaxy workflows enables researchers to perform more efficient analyses. For example, Cumbo et al. 2024^47^ employed machine learning models for identifying potential biomarkers that can distinguish between healthy and diseased states on public metagenomic samples collected from patients affected by colorectal cancer in a case/control scenario.

Clinical metaproteomics holds immense potential for expanding the understanding of host-microbe interactions underlying human disease in microbiome research. However, challenges persist in this field, with the foremost one being the characterisation of microbial proteins present in low abundance relative to host proteins. Other hurdles faced by researchers stem from the use of very large protein sequences databases in peptide and protein identification from mass spectrometry (MS) data in addition to performing rigorous analyses of identified peptides, including taxonomic and functional assignments as well as statistical analysis to attain differentially abundant peptides. Galaxy clinical metaproteomics workflows address such challenges. These workflows have been applied to diverse sample types, including nasopharyngeal swab from COVID-19 patients, bronchoalveolar lavage samples from cystic fibrosis patients, and the development of biomarker panels for early detection of ovarian cancer^48–50^.

In environmental research, metaproteomics workflow in Galaxy has proven effective for studying microbial communities in soils and aquatic ecosystems. For instance, a recent study of North Atlantic Ocean samples^51^ compared workflows for the taxonomic and functional analyses of mass spectrometry data using Galaxy tools like MaxQuant (v.1.6.17.0)^52^ (RRID:SCR_014485) and SearchGUI (v.3.3.10.1), which included multiple search algorithms (X!Tandem, MS-GF+, and Comet) and PeptideShaker (v.1.16.36). The results underscored the importance of robust and reproducible workflows for analysing environmental samples. Furthermore, Galaxy allows easy sharing of the workflows, which is of fundamental importance in multi-laboratory analyses and big data sharing. These metaproteomics workflows are also used by the members of the Metaproteomics Initiative (https://metaproteomics.org/).

Building on this metaproteomics foundation, Schiml et al. 2023^37^ extended the approach to meta-omics in their study of a cellulose-degrading microbial consortium from an industrial biogas reactor in Norway. They employed metagenomics to recover metagenome-assembled genomes (MAGs), including *Hungateiclostridium thermocellum, Thermoclostridium stercorarium*, and multiple heterogenic strains affiliated to *Coprothermobacter proteolyticus*. The predicted genes from the metagenomics analysis have been used to construct databases for mRNA and protein identification and quantification. Metatranscriptomics reveals the functional potential of these microbes, while metaproteomics identifies expressed proteins and active metabolic pathways, linking them to specific MAGs. Integrating these meta-omics techniques within interlaced workflows provided a comprehensive view of microbial interactions and their roles in biomass degradation, with annotations for carbohydrate-active enzymes (CAZymes), as well as KEGG annotations and pathways.

Another example from environmental microbiology is the workflow developed by Péguilhan et al. 2023^53^, which examined microbial gene expression in clouds and clear air masses at high altitudes. Differential gene expression analysis revealed that multiple biological processes are triggered in clouds, including fungal spore germination, energy metabolism, autophagy and starvation. This provided unprecedented insights into the functioning of microorganisms in these rarely explored environments. The choice of databases for taxonomic affiliation was extended to include environmental datasets, and the workflow involved a specific gene catalogue created by merging all the metagenomics sequences of the study into a non-redundant annotated database, used as a reference database for differential gene expression analysis using the metatrascriptomics data. This study highlights the flexibility of Galaxy tools and their suitability to explore microbial ecology in the environment.

In addition to its utility in research, Galaxy can be a powerful tool for citizen education and broadening participation in science. The BeerDEcoded project, led by the Street Science Community, engages participants from diverse backgrounds to analyse beer samples’ microbiomes (https://streetscience.community/). Galaxy’s user-friendly interface and workflows enabled high school pupils to explore microbiome analysis while learning the principles of open science. Similarly, the BioDIGS project, part of the Genomic Data Science Community Network (GDSCN)^54^, empowers scientists from underserved institutions across the United States to investigate the microbial life of their local environments (https://biodigs.org/). In this regard, Galaxy is also utilized by the non-profit organization SPUN (https://www.spun.earth/) in their global effort to map mycorrhizal fungal communities.

Through Galaxy, participants process and analyse DNA sequencing data from samples collected in their communities, contributing to global efforts to understand microbial diversity. All these use cases underscore microGalaxy’s capability to address complex microbial research challenges across diverse domains.

#### Future vision of the microGalaxy community

As microbial research evolves, so must the resources available to analyse generated data; microGalaxy inevitably needs to evolve alongside these developments. Several key future directions present opportunities for expanding the platform’s capabilities, improving multi-omics data integration, and addressing emerging challenges in microbial research.

While Galaxy already offers robust support for SARS-Cov-2^55^ and MPOX analyses, expanding its capabilities to better handle viruses, archaea, and eukaryotes will be crucial in addressing gaps in microbial research. Indeed, despite their important roles in microbial communities, non-bacterial entities are often underrepresented in many ecological studies due to the domination of prokaryotic signals^56^. Supporting these data more comprehensively will open up new research opportunities, particularly in ecosystems where these entities play significant roles, such as in extreme environments or host-associated microbiomes.

Furthermore, expanding microGalaxy’s multi-omics support, particularly through holo-omics approaches for host-microbiome integration, would provide a more holistic understanding of microbial dynamics. By integrating genomics, transcriptomics, proteomics, and metabolomics, researchers can gain deeper insights into the functional relationships between microbes and their hosts, further enhancing the utility of microGalaxy in complex biological studies.

Integrating other omics tools and software is one significant avenue for expanding microGalaxy’s capabilities. For instance, integrating Anvi’o^8^, a tool designed for visualising and analysing large-scale microbial genomes, or Nextstrain^57^ with Galaxy would significantly enhance the microGalaxy’s ability to conduct advanced genomic analyses, such as pangenomics and phylogenomics. The Anvi’o’s integration would enable users to combine Galaxy’s workflows with Anvi’o’s powerful visualisation tools, fostering more in-depth exploration of complex microbial communities. Additionally, integrating differential expression analysis tools, such as MTXmodel for metatranscriptomics, would provide a standardised approach for integrating metatranscriptomic and metagenomic data. This would enable researchers to more effectively study microbial gene expression dynamics in various environmental and host-associated contexts.

An important opportunity for microGalaxy’s development is its capacity to enable federated data analysis. Collaborations with platforms like MGnify, which offer large-scale datasets from diverse microbial communities, could allow researchers to perform cross-platform studies. As exemplified by integrating the MGnify amplicon workflow in Galaxy, such collaborations enhance the accessibility of microbial datasets, facilitating large-scale studies across diverse environments and systems.

Similar to the approach taken in ABRomics, microGalaxy workflows are being integrated into Bioinformatics Resource Centers (BRCs) for Infectious Diseases resources. BRC Analytics (https://brc-analytics.org/), provides a comprehensive platform for exploring and interpreting genomic annotations and functional insights into disease-causing organisms and their carriers, using Galaxy and its Application Programming Interface (API). By incorporating microGalaxy workflows, BRC Analytics users can seamlessly analyse microbial genomic, transcriptomic, and multi-omic datasets within a well-established bioinformatics framework, promoting collaboration across disciplines and increasing the reach and impact of microbial research efforts. This integration would also make advanced analysis tools available to a broader research community, improving overall efficiency and ensuring data interoperability in large-scale, cross-disciplinary studies.

As illustrated by ABRomics, clinical metaproteomics, or integrative meta-omics workflows, microGalaxy has the potential to make significant contributions to public health and environmental monitoring, particularly in the fields of One Health and biodiversity surveillance. In One Health initiatives, which focus on human, animal, and environmental health interconnectedness, microGalaxy could support studies investigating antimicrobial resistance (AMR) and zoonotic pathogens. Additionally, biodiversity surveillance—especially in monitoring microbial communities in soils, oceans, and other ecosystems—can benefit from integrating Galaxy’s workflows with ecological and environmental datasets. By combining genomic data with environmental variables, researchers can gain insights into how microbial diversity responds to global changes, improving our understanding of ecosystem health and resilience.

As more microbial data are generated, managing computational resources to process this data effectively will be a critical challenge. Tools that predict computational resources based on input datasets requirements will be essential in addressing this challenge. By allowing Galaxy administrators to dynamically allocate the computational needs of the tools with the Total Perspective Vortex (https://github.com/galaxyproject/total-perspective-vortex), microGalaxy can ensure that researchers can run large-scale analyses on available infrastructure without overloading resources. This feature will improve everyone’s fair access to Galaxy and high-performance computing resources in microbial research.

In summary, microGalaxy is more than just a comprehensive set of tools for microbial research. It provides an essential community-driven infrastructure that supports collaboration and the integration of diverse analytical workflows. By prioritising reproducibility, accessibility, and scalability, microGalaxy is in a unique position to advance the field of microbial data analysis and enhance our understanding of microbial life.

### Methods

#### Galaxy framework and microGalaxy Lab

At the core of microGalaxy is the Galaxy framework^12^, a robust and widely adopted platform that enables accessible and reproducible bioinformatics analyses. microGalaxy extends the Galaxy framework using a community-specific interface, called Galaxy Lab. A Galaxy Lab provides users with a focused workspace that retains all the benefits of the Galaxy ecosystem, including accessibility, scalability, and reproducibility but is tailored specifically to the community needs. Focused on microbial research, microGalaxy facilitates access to a comprehensive set of tool suites, workflows, and training materials. It ensures that everyone at any level of expertise can efficiently perform analyses, thus fostering a supportive and inclusive environment for microbial research.

The microGalaxy Lab is deployed on public Galaxy servers, such as usegalaxy.eu, further enhancing its accessibility and usability. These servers host the microGalaxy Lab as a dedicated subdomain, ensuring streamlined access to its tools and workflows while leveraging the robust computational resources provided by the computing infrastructures hosting these servers. With microGalaxy Lab available on several major public Galaxy instances, researchers worldwide can access cutting-edge microbiological analysis capabilities without needing local installations or specialised hardware. Although not as a separate interface, the microGalaxy tools are also available on several Galaxy servers (e.g. usegalaxy.org or usegalaxy.fr, Supplementary Figure 3A).

All resources supporting the microGalaxy Lab, including its customised interface, tools, and workflows, are curated and stored within the Galaxy CoDex GitHub repository (https://github.com/usegalaxy-eu/galaxy-codex). The Galaxy CoDex is a centralised repository, ensuring versioning, and documentation of microGalaxy components.

#### Community-Curated Tools

The tools in microGalaxy are sourced from the Galaxy ToolShed^58^ (https://toolshed.g2.bx.psu.edu), an extensive repository hosting Galaxy tool wrappers. These tool wrappers provide the integration layer between a command of external software and the Galaxy platform, defining inputs/outputs (including their formats) and parameters. These tool wrappers are developed and maintained by the Galaxy community and groups such as the Intergalactic Utilities Commission (IUC) (https://galaxyproject.org/iuc/), with their source code managed in GitHub repositories. Planemo^59^, a Software Development Kit (SDK) for Galaxy tools, is instrumental in the development process. It is a command-line utility to assist in creating, testing, and validating Galaxy tool wrappers, promoting consistency and high-quality standards across the platform. Once developed and approved by the community, tools are stored in the ToolShed for easy access and installation on any Galaxy server. The Galaxy ToolShed is, in this way, an App Store for the Galaxy community.

Any tool dependencies are resolved using packages or containers available through Bioconda^60^ or Biocontainers^61^. When the dependencies are updated, tools maintained by the IUC or other community tool repositories undergo a semi-automated updating process. Once approved by a community member, the updated tool becomes publicly available on ToolShed and is automatically updated on the major Galaxy servers where they are installed. This process ensures that users have access to the latest versions of tools. At the same time, legacy versions are retained to support reproducibility, enabling researchers to repeat analyses even as tools evolve.

Tools in the Galaxy ToolShed are organised into individual entries (a single command) or grouped into tool suites (a set of commands from a software). microGalaxy hosts a subset of these tool suites ready to be used within the microGalaxy Lab. This ensures a streamlined experience for users, making the tools readily available without requiring additional configuration.

#### Workflows

Workflows are at the core of microGalaxy. microGalaxy workflows exist at four levels of development, which provide varying degrees of accessibility, adherence to best practices, and maintenance: (i) Publicly shared workflow: These workflows, tagged with #microGalaxy, are readily accessible to the research community, available across public Galaxy instances and offer a starting point for users seeking to conduct microbial data analysis, (ii) Workflows in WorkflowHub^30^: Workflows published in WorkflowHub, a workflow registry, are annotated with information such as creator information and licensing. These workflows may not include test data, but they offer documented and structured analysis workflows for microbial research, (iii) Workflows in WorkflowHub related to tutorials: Tutorials on the Galaxy Training Network are recommended to be supported by workflows. These workflows adhere to Galaxy’s best practices, i.e. which include clear annotations, proper input and output parameter definitions, and formal documentation (such as creator information and licensing) and include test data. (iv) IWC-Validated Workflows on WorkflowHub and Dockstore: A subset of workflows available through WorkflowHub has been further curated through the Intergalatic Workflow Commission (IWC) to adhere to Galaxy’s best practices, include test data, and support state-of-the-art analyses. Stored in the IWC GitHub repository, they are supported by a semi-automated updating system: when a Galaxy tool used in the workflow is updated, a continuous integration pipeline tests the workflow with the updated tool version. Once approved by a community member, the updated workflow becomes publicly available on WorkflowHub and Dockstore and the major Galaxy instances. In addition, Galaxy users can easily import workflows from WorkflowHub directly into their Galaxy server, enabling seamless integration into their analysis pipelines. This process ensures that microGalaxy workflows remain up-to-date and reproducible, making them an invaluable resource for microbial research.

#### Training materials

Training is critical to equipping users with the knowledge needed to fully leverage the platform’s capabilities. The global Galaxy community has developed a comprehensive suite of over 400 tutorials, which are available on the Galaxy Training Network (GTN)^41,42^. A subset of these tutorials, tagged with the term “microGalaxy” (https://training.galaxyproject.org/training-material/search2?query=microgalaxy), is dedicated to microbial data analysis. These tutorials cover various topics, from basic genomics to more complex analyses like metaproteomics (https://gxy.io/GTN:T00221) which allows the user to learn how to match mass spectrometry data to peptide sequences, perform taxonomy and functional analysis, and visualize metaproteomics data. They are designed to support learners of varying expertise levels, from beginners to experienced researchers.

The GTN tutorials are continually updated to incorporate the state-of-the-art tools, workflows, and methodologies, ensuring users can access current information. Many tutorials are structured into learning pathways, which provide a step-by-step progression of topics, guiding learners through increasingly complex analyses. These learning pathways are designed to build foundational knowledge while advancing skills for handling cutting-edge microbial data.

In addition to written tutorials, the GTN offers recordings as an interactive and flexible learning method. These videos are accompanied by manually curated captions, making them more accessible to a broader audience, including those who may prefer visual or translated content.

microGalaxy also offers a range of training events, such as workshops, hackathons, and online seminars. These events allow users to learn, engage directly with experts, share experiences, and deepen their understanding of the platform’s capabilities.

#### Resource aggregation and annotation

Resource annotation and ontologies are essential for ensuring consistency and improving discoverability across the microGalaxy platform. To achieve this, Galaxy employs the EDAM ontology^62^, a structured vocabulary for bioinformatics concepts, to categorise tools, workflows, and training materials. This ontology-based approach ensures consistent descriptions across the platform, streamlining resource discovery and enabling users to quickly find resources tailored to their research needs.

All Galaxy resources are aggregated into the Galaxy CoDex (https://github.com/galaxyproject/galaxy_codex), a comprehensive catalog that integrates metadata from the Galaxy ecosystem, bio.tools, and WorkflowHub. The CoDex enables the creation of lists and widgets that can be embedded into websites, offering seamless access to up-to-date tools, workflows, and training materials.

During the aggregation process, missing tool annotations were identified; hence, an annotation process was essential for resources lacking EDAM annotations, particularly tools not linked to external databases like bio.tools. The microGalaxy community has extensively worked to improve resource annotations during hackathons^44,45^ and other collaborative activities. As a result, over 300 tools have been annotated, and more than 30 tutorials have been enriched with relevant EDAM terms. These efforts have enhanced the categorisation and discoverability of resources within microGalaxy, making it easier for researchers to locate the appropriate resources for their analyses.

#### Survey and use cases

A survey was conducted between March and September 2023 to assess the needs, preferences, and challenges of the microbial research community. The survey aimed to gather insights into how researchers interact with Galaxy for microbial research and identify areas for improvement.

The survey was developed by the microGalaxy community and was distributed online via the Galaxy Project website, mailing lists, and social media channels. It consisted of 16 questions (Supplementary Document 1) across several categories including (i) research focus and community demographics, (ii) tools and workflows, (iii) training and support, and (iv) future developments. The survey included multiple-choice, Likert scale, and open-ended questions to allow collecting both quantitative and qualitative information.

Participation was voluntary, and the purpose of the study was explained at the top of the survey.

A total of 130 researchers (Supplementary Table 2) participated in the survey, representing a diverse range of geographical locations, institutions, and research domains. Quantitative responses were analysed using descriptive statistics to summarise trends and identify prevalent themes. Qualitative responses to open-ended questions were coded and analysed thematically to extract insights into specific challenges and recommendations.

Survey participants who indicated willingness to be contacted were sent a follow-up invitation, including a structured document (Supplementary Document 2) to collect detailed information about their use cases. This document included (i) research objectives and questions, (ii) methods employed, including experimental techniques, data generation approaches, and analysis pipelines, and (iii) tools and workflows used within and outside Galaxy.

The 21 use cases were collected and anonymised (Supplementary Table 5). The compelling use cases were elaborated upon in the main text of this study, and all authors were invited to contribute to the microGalaxy manuscript preparation.

#### Citation Extraction and Annotation

To analyse the impact of Galaxy on microbial research, citations of the major Galaxy papers were extracted via Python (v 3.8.19) within a Jupyter Notebook (v 1.0.0) (RRID:SCR_018315). The 8 major publications of the Galaxy Project were extracted from the Galaxy Project’s Google Scholar profile (https://scholar.google.com/citations?hl=en&user=3tSiRGoAAAAJ) using scholarly (v 1.7.11)^63^. These publications and their citations were then retrieved on Semantic Scholar^64^ via its Application Programming Interface (API) using requests (v 2.32.3). The collected data included the publication years, titles, and abstracts.

Citations were annotated as microbial-related if their titles or abstracts contained at least one of the 23 predefined keywords relevant to microbial research (“bacteri”, “prokaryot”, “microb”, “pathogen”, “virus”, “phage”, “archae”, “flora”, “microecology”, “microorganism”, “micro-organism”, “metabarcod”, “16s”, “18s”, “amplicon”, “metataxonom”, “metagenom”, “metatranscriptom”, “metaproteom”, “multi-locus sequence typing”, “multilocus sequence typing”, “mlst”, “otu”). These keywords were chosen to capture diverse aspects of microbial studies, including terms related to targeted organisms (e.g., “bacteria,” “microbiome”), technical objectives (e.g., “functional analysis,” “MAGs”), and methodologies (e.g., “metagenomics,” “metaproteomics”). Each microbial-related citation was further categorised into three dimensions given keywords in their titles and abstracts to enable a detailed analysis of the research themes addressed in the citing papers: (i) Targeted Organisms (Bacteria, Virus, Archaea, Eukaryote, Microbiome, Pathogen), (ii) Technical Targets (Isolate, Community (taxonomy) profiling, Functional analysis, Interactome, AMR, MAGs, Gene identification / Biomarker, SNP, (M)LST, Annotation, Variant, Comparative analysis), and (iii) Methods (Metabarcoding, (Meta)genomics, Metagenomics, (Meta)transcriptomics, Metatranscriptomics, (Meta)proteomics, Metaproteomics, Metabolomics, Imaging).

#### Data visualisation

To analyse and visualise data, a set of R Markdown (https://github.com/rstudio/rmarkdown) scripts were created and hosted in the microGalaxy article GitHub repository. These scripts streamlined the generation of figures and statistical analyses presented in this study, allowing for reproducibility and updates as new data become available. Each script ingests tables generated by Galaxy CoDex, filtered for the microGalaxy community, containing metadata and EDAM annotations for tools, training materials, and workflows. This setup enables the calculation of key metrics such as total counts, distribution across EDAM terms, and user feedback statistics.

data.table (v1.14.8) and tidyr (v1.1.3) libraries are used for data manipulation, with stringr (v1.5.0) aiding in text processing. Visualisations are primarily created with ggplot2 (v3.4.4), while ggrepel (v0.9.3), ggtext (v0.1.2), and ggh4x (v0.2.5) are used for labelling, rich text, and facet customisation, respectively. Colorspace, and paletteer (v2.1–0) ensured accessible colour schemes, packcircles (v0.3.5) handled circle-packing layouts, and shadowtext (v0.1.2) improved label readability. Additionally, extrafont (v0.18) is used to manage custom fonts. All analyses are run on R version 4.3.1.

## Supplementary Material

Supplement 1

Supplement 2

Supplement 3

Supplement 4

Supplement 5

Supplement 6

Supplement 7

Supplement 8

1

## References

[1] Di Bella J. M., Bao Y., Gloor G. B., Burton J. P. & Reid G. High throughput sequencing methods and analysis for microbiome research. J. Microbiol. Methods 95, 401–414 (2013). 24029734 10.1016/j.mimet.2013.08.011

[2] Reuter J. A., Spacek D. V. & Snyder M. P. High-Throughput Sequencing Technologies. Mol. Cell 58, 586–597 (2015). 26000844 10.1016/j.molcel.2015.05.004 PMC4494749

[3] Wilkinson M. D. The FAIR Guiding Principles for scientific data management and stewardship. Sci. Data 3, 160018 (2016). 26978244 10.1038/sdata.2016.18 PMC4792175

[4] One Health High-Level Expert Panel (OHHLEP) One Health: A new definition for a sustainable and healthy future. PLOS Pathog. 18, e1010537 (2022). 35737670 10.1371/journal.ppat.1010537 PMC9223325

[5] Richardson L. MGnify: the microbiome sequence data analysis resource in 2023. Nucleic Acids Res. 51, D753–D759 (2023). 36477304 10.1093/nar/gkac1080 PMC9825492

[6] Keegan K. P., Glass E. M. & Meyer F. MG-RAST, a Metagenomics Service for Analysis of Microbial Community Structure and Function. in Microbial Environmental Genomics (MEG) (eds. Martin F. & Uroz S.) vol. 1399 207–233 (Springer New York, New York, NY, 2016). 10.1007/978-1-4939-3369-3_13 26791506

[7] McIver L. J. bioBakery: a meta’omic analysis environment. Bioinformatics 34, 1235–1237 (2018). 29194469 10.1093/bioinformatics/btx754 PMC6030947

[8] Eren A. M. Community-led, integrated, reproducible multi-omics with anvi’o. Nat. Microbiol. 6, 3–6 (2020). 10.1038/s41564-020-00834-3 PMC8116326 33349678

[9] Arkin A. P. KBase: The United States Department of Energy Systems Biology Knowledgebase. Nat. Biotechnol. 36, 566–569 (2018). 29979655 10.1038/nbt.4163 PMC6870991

[10] Olson R. D. Introducing the Bacterial and Viral Bioinformatics Resource Center (BV-BRC): a resource combining PATRIC, IRD and ViPR. Nucleic Acids Res. 51, D678–D689 (2023). 36350631 10.1093/nar/gkac1003 PMC9825582

[11] Cheng K. MetaLab: an automated pipeline for metaproteomic data analysis. Microbiome 5, 157 (2017). 29197424 10.1186/s40168-017-0375-2 PMC5712144

[12] The Galaxy Community. The Galaxy platform for accessible, reproducible, and collaborative data analyses: 2024 update. Nucleic Acids Res. 52, W83–W94 (2024). 38769056 10.1093/nar/gkae410 PMC11223835

[13] Nassar L. R. The UCSC Genome Browser database: 2023 update. Nucleic Acids Res. 51, D1188–D1195 (2023). 36420891 10.1093/nar/gkac1072 PMC9825520

[14] Sayers E. W. Database resources of the national center for biotechnology information. Nucleic Acids Res. 50, D20–D26 (2022). 34850941 10.1093/nar/gkab1112 PMC8728269

[15] Burgin J. The European Nucleotide Archive in 2022. Nucleic Acids Res. 51, D121–D125 (2023). 36399492 10.1093/nar/gkac1051 PMC9825583

[16] Blomer J., Buncic P. & Fuhrmann T. CernVM-FS: delivering scientific software to globally distributed computing resources. in Proceedings of the first international workshop on Network-aware data management 49–56 (Association for Computing Machinery, New York, NY, USA, 2011). doi:10.1145/2110217.2110225.

[17] Chen S., Zhou Y., Chen Y. & Gu J. fastp: an ultra-fast all-in-one FASTQ preprocessor. Bioinformatics 34, i884–i890 (2018). 30423086 10.1093/bioinformatics/bty560 PMC6129281

[18] Martin M. Cutadapt removes adapter sequences from high-throughput sequencing reads. EMBnet.journal 17, 10–12 (2011).

[19] Seemann T. Prokka: rapid prokaryotic genome annotation. Bioinformatics 30, 2068–2069 (2014). 24642063 10.1093/bioinformatics/btu153

[20] Bankevich A. SPAdes: A New Genome Assembly Algorithm and Its Applications to Single-Cell Sequencing. J. Comput. Biol. 19, 455–477 (2012). 22506599 10.1089/cmb.2012.0021 PMC3342519

[21] Schloss P. D. Reintroducing mothur: 10 Years Later. Appl. Environ. Microbiol. 86, e02343–19 (2020). 31704678 10.1128/AEM.02343-19 PMC6952234

[22] Wood D. E., Lu J. & Langmead B. Improved metagenomic analysis with Kraken 2. Genome Biol. 20, 257 (2019). 31779668 10.1186/s13059-019-1891-0 PMC6883579

[23] Breitwieser F. P. & Salzberg S. L. Pavian: interactive analysis of metagenomics data for microbiome studies and pathogen identification. Bioinformatics 36, 1303–1304 (2020). 31553437 10.1093/bioinformatics/btz715 PMC8215911

[24] Bik H. M. & Inc P. I. Phinch: An interactive, exploratory data visualization framework for–Omic datasets. 009944 Preprint at 10.1101/009944 (2014).

[25] McMurdie P. J. & Holmes S. Shiny-phyloseq: Web application for interactive microbiome analysis with provenance tracking. Bioinformatics 31, 282–283 (2015). 25262154 10.1093/bioinformatics/btu616 PMC4287943

[26] Lewis S. Apollo: a sequence annotation editor. Genome Biol. 3, research0082.1 (2002). 10.1186/gb-2002-3-12-research0082 PMC151184 12537571

[27] Gangiredla J. GalaxyTrakr: a distributed analysis tool for public health whole genome sequence data accessible to non-bioinformaticians. BMC Genomics 22, 114 (2021). 33568057 10.1186/s12864-021-07405-8 PMC7877046

[28] Di Tommaso P. Nextflow enables reproducible computational workflows. Nat. Biotechnol. 35, 316–319 (2017). 28398311 10.1038/nbt.3820

[29] Mölder F. Sustainable data analysis with Snakemake. F1000Research 10, 33 (2021). 34035898 10.12688/f1000research.29032.1 PMC8114187

[30] Gustafsson O. J. R. WorkflowHub: a registry for computational workflows. Preprint at 10.48550/ARXIV.2410.06941 (2024). PMC12095652 40399296

[31] Yuen D. The Dockstore: enhancing a community platform for sharing reproducible and accessible computational protocols. Nucleic Acids Res. 49, W624–W632 (2021). 33978761 10.1093/nar/gkab346 PMC8218198

[32] Nasr E., Henger A., Grüning B., Zierep P. & Batut B. PathoGFAIR: a collection of FAIR and adaptable (meta)genomics workflows for (foodborne) pathogens detection and tracking. Preprint at 10.1101/2024.06.26.600753 (2024). PMC12466118 41004266

[33] Barsnes H. & Vaudel M. SearchGUI: A Highly Adaptable Common Interface forProteomics Search and de Novo Engines. J. Proteome Res. 17, 2552–2555 (2018). 29774740 10.1021/acs.jproteome.8b00175

[34] Vaudel M. PeptideShaker enables reanalysis of MS-derived proteomics data sets. Nat. Biotechnol. 33, 22–24 (2015). 25574629 10.1038/nbt.3109

[35] Vande Moortele T. Unipept in 2024: Expanding Metaproteomics Analysis with Support for Missed Cleavages, Semi-Tryptic and Non-Tryptic Peptides. Preprint at 10.1101/2024.09.26.615136 (2024). 39792626

[36] The Gene Ontology Consortium The Gene Ontology knowledgebase in 2023. Genetics 224, iyad031 (2023). 10.1093/genetics/iyad031 PMC10158837 36866529

[37] Schiml V. C. Integrative meta-omics in Galaxy and beyond. Environ. Microbiome 18, 56 (2023). 37420292 10.1186/s40793-023-00514-9 PMC10329324

[38] Peters K. PhenoMeNal: processing and analysis of metabolomics data in the cloud. GigaScience 8, giy149 (2019). 10.1093/gigascience/giy149 PMC6377398 30535405

[39] Giacomoni F. Workflow4Metabolomics: a collaborative research infrastructure for computational metabolomics. Bioinforma. Oxf. Engl. 31, 1493–1495 (2015). 10.1093/bioinformatics/btu813 PMC4410648 25527831

[40] Davidson R. L., Weber R. J. M., Liu H., Sharma-Oates A. & Viant M. R. Galaxy-M: a Galaxy workflow for processing and analyzing direct infusion and liquid chromatography mass spectrometry-based metabolomics data. GigaScience 5, 10 (2016). 26913198 10.1186/s13742-016-0115-8 PMC4765054

[41] Hiltemann S. Galaxy Training: A powerful framework for teaching! PLOS Comput. Biol. 19, e1010752 (2023). 36622853 10.1371/journal.pcbi.1010752 PMC9829167

[42] Batut B. Community-Driven Data Analysis Training for Biology. Cell Syst. 6, 752–758.e1 (2018). 29953864 10.1016/j.cels.2018.05.012 PMC6296361

[43] Rasche H. Training Infrastructure as a Service. GigaScience 12, giad048 (2023). 10.1093/gigascience/giad048 PMC10316688 37395629

[44] Zierep P. How to increase the findability, visibility, and impact of Galaxy tools for your scientific community. Preprint at 10.37044/osf.io/qjbxc (2024).

[45] Batut B. How to improve the annotation of Galaxy resources? Outcomes of an online hackathon for improving the annotation of Galaxy resources for microbial data resources. Preprint at 10.37044/osf.io/s7tru (2024).

[46] Merdan O. Investigation of the Defective Growth Pattern and Multidrug Resistance in a Clinical Isolate of Candida glabrata Using Whole-Genome Sequencing and Computational Biology Applications. Microbiol. Spectr. 10, e00776–22 (2022). 35867406 10.1128/spectrum.00776-22 PMC9430859

[47] Cumbo F., Truglia S., Weitschek E. & Blankenberg D. Feature selection with vector-symbolic architectures: a case study on microbial profiles of shotgun metagenomic samples of colorectal cancer. Preprint at 10.1101/2024.11.18.624180 (2024). PMC12018301 40269516

[48] Bihani S. Metaproteomic Analysis of Nasopharyngeal Swab Samples to Identify Microbial Peptides in COVID-19 Patients. J. Proteome Res. 22, 2608–2619 (2023). 37450889 10.1021/acs.jproteome.3c00040

[49] Do K. A novel clinical metaproteomics workflow enables bioinformatic analysis of host-microbe dynamics in disease. mSphere 9, e00793–23 (2024). 38780289 10.1128/msphere.00793-23 PMC11332332

[50] Kruk M. E. An integrated metaproteomics workflow for studying host-microbe dynamics in bronchoalveolar lavage samples applied to cystic fibrosis disease. mSystems 9, e00929–23 (2024). 10.1128/msystems.00929-23 PMC11264604 38934598

[51] Saito M. A. Results from a multi-laboratory ocean metaproteomic intercomparison: effects of LC-MS acquisition and data analysis procedures. Biogeosciences 21, 4889–4908 (2024).

[52] Cox J. & Mann M. MaxQuant enables high peptide identification rates, individualized p.p.b.-range mass accuracies and proteome-wide protein quantification. Nat. Biotechnol. 26, 1367–1372 (2008). 19029910 10.1038/nbt.1511

[53] Péguilhan R. Clouds influence the functioning of airborne microorganisms. EGUsphere 1–27 (2024) doi:10.5194/egusphere-2024-2338.

[54] Network T. G. D. S. C. Diversifying the genomic data science research community. Genome Res. 32, 1231–1241 (2022). 35858750 10.1101/gr.276496.121 PMC9341509

[55] Maier W. Ready-to-use public infrastructure for global SARS-CoV-2 monitoring. Nat. Biotechnol. 39, 1178–1179 (2021). 34588690 10.1038/s41587-021-01069-1 PMC8845060

[56] Bazant W., Blevins A. S., Crouch K. & Beiting D. P. Improved eukaryotic detection compatible with large-scale automated analysis of metagenomes. Microbiome 11, 72 (2023). 37032329 10.1186/s40168-023-01505-1 PMC10084625

[57] Hadfield J. Nextstrain: real-time tracking of pathogen evolution. Bioinformatics 34, 4121–4123 (2018). 29790939 10.1093/bioinformatics/bty407 PMC6247931

[58] Blankenberg D. Dissemination of scientific software with Galaxy ToolShed. Genome Biol. 15, 403 (2014). 25001293 10.1186/gb4161 PMC4038738

[59] Bray S. The Planemo toolkit for developing, deploying, and executing scientific data analyses in Galaxy and beyond. Genome Res. 33, 261–268 (2023). 36828587 10.1101/gr.276963.122 PMC10069471

[60] The Bioconda Team Bioconda: sustainable and comprehensive software distribution for the life sciences. Nat. Methods 15, 475–476 (2018). 29967506 10.1038/s41592-018-0046-7 PMC11070151

[61] Da Veiga Leprevost F. BioContainers: an open-source and community-driven framework for software standardization. Bioinformatics 33, 2580–2582 (2017). 28379341 10.1093/bioinformatics/btx192 PMC5870671

[62] Ison J. EDAM: an ontology of bioinformatics operations, types of data and identifiers, topics and formats. Bioinformatics 29, 1325–1332 (2013). 23479348 10.1093/bioinformatics/btt113 PMC3654706

[63] Cholewiak S., Ipeirotis P., Silva V. & Kannawadi A. scholarly. Zenodo 10.5281/ZENODO.7542349 (2023).

[64] Kinney R. The Semantic Scholar Open Data Platform. Preprint at 10.48550/ARXIV.2301.10140 (2023).

