# The Microbiology Galaxy Lab: A community-driven gateway to tools, workflows, and training for reproducible and FAIR analysis of microbial data

**DOI:** 10.1101/2024.12.23.629682

**Published:** 2025-03-26

**Authors:** Engy Nasr, Pierre Amato, Matthias Bernt, Anshu Bhardwaj, Daniel Blankenberg, Daniela Brites, Fabio Cumbo, Katherine Do, Emanuele Ferrari, Timothy J. Griffin, Björn Grüning, Saskia Hiltemann, Cameron Hyde, Pratik Jagtap, Subina Mehta, Kimberly Métris, Saim Momin, Gareth R Price, Asime Oba, Christina Pavloudi, Nikos Pechlivanis, Raphaëlle Péguilhan, Fotis Psomopoulos, Nedeljka Rosic, Michael C. Schatz, Valerie Claudia Schiml, Cléa Siguret, Nicola Soranzo, Andrew Stubbs, Peter van Heusden, Mustafa Vohra, microGalaxy Community, Paul Zierep, Bérénice Batut

**Affiliations:** 1 Bioinformatics Group, Department of Computer Science, University of Freiburg, Georges-Koehler-Allee 106, D-79110 Freiburg, Germany; 2 Université Clermont Auvergne, CNRS, Laboratoire Microorganismes: Génomes et Environnement (LMGE), F-63000, Clermont-Ferrand, France; 3 Department of Computational Biology and Chemistry, Helmholtz Centre for Environmental Research - UFZ, Permoserstraße 15, Leipzig, D-04318, Germany; 4 CSIR-Institute of Microbial Technology, Chandigarh, and Academy of Scientific & Innovative Research (AcSIR), Delhi, India; 5 Center for Computational Life Sciences, Cleveland Clinic, Cleveland, Ohio, USA; 6 Swiss Tropical and Public Health Institute, University of Basel, Basel, Switzerland; 7 University of Minnesota, Minneapolis, MN, USA; 8 National Research Council of Italy – Water Research Institute (CNR-IRSA) Molecular Ecology Group (MEG), Verbania, Italy; 9 University of Freiburg, Faculty of Chemistry and Pharmacy, Freiburg, Germany; 10 Australian BioCommons, The University of Melbourne, Melbourne, Victoria, 3010, Australia; 11 Department of Genetics and Biochemistry, Clemson University, Clemson, South Carolina 29634, USA; 12 The University of Queensland, Brisbane, Queensland, 4152, Australia; 13 Queensland Cyber Infrastructure Foundation, Brisbane, Queensland, 4152, Australia; 14 University of Maiduguri, Maiduguri, Nigeria; 15 European Marine Biological Resource Centre (EMBRC-ERIC), Paris, France; 16 Institute of Applied Biosciences, Centre for Research and Technology Hellas, Thermi, 57001, Thessaloniki, Greece; 17 Department of Chemical and Biochemical Engineering, Technical University of Denmark, DK-2800 Kgs. Lyngby, Denmark; 18 Faculty of Health, Southern Cross University, Gold Coast, QLD, Australia; 19 Department of Computer Science, Johns Hopkins University, Baltimore, Maryland 21218, USA; 20 The Protein Engineering and Proteomics Group (PEP), Faculty of Chemistry, Biotechnology and Food Science, Norwegian University of Life Sciences, Elizabeth Stephansens vei 15, 1433 Ås, Norway; 21 IFB-core, Institut Français de Bioinformatique, CNRS, INSERM, INRAE, CEA, Evry, France; 22 Plateforme AuBi, Mésocentre Clermont-Auvergne, Université Clermont Auvergne, Aubière, France; 23 Earlham Institute, Norwich, UK; 24 Department of Pathology and Clinical Bioinformatics, Erasmus MC Cancer Institute, Erasmus MC, Rotterdam, Netherlands; 25 South African Medical Research Council Bioinformatics Unit, South African National Bioinformatics Institute, University of the Western Cape; 26 Department of Medical Laboratory Science, Lovely Professional University, Punjab 144411, India; 27 Department of Microbiology, Shri Vinoba Bhave Civil Hospital, Silvassa 396230, India

## Abstract

Microbial research generates vast and complex data from diverse omics technologies, demanding innovative analytical solutions. The microGalaxy community addresses these needs with the Microbiology Lab, a user-friendly platform that integrates 290+ tool suites and 85+ curated workflows for microbial analyses, including taxonomic profiling, assembly, annotation, and functional analysis. Hosted on several public Galaxy servers (microbiology.usegalaxy.org, .eu, .org.au, .fr), it supports workflow creation & customization, sharing, and updates across public and private Galaxy servers, ensuring flexibility and reproducibility. The platform also offers 35+ tutorials, 15+ instructional videos, and structured learning pathways, empowering researchers to conduct advanced analyses. Backed by a community-driven approach, the Microbiology Galaxy Lab ensures up-to-date tools and workflows through testing and semi-automatic updates to meet global research demands. With its focus on rapid workflow prototyping and high-throughput processing, the Microbiology Galaxy Lab provides scalable resources for researchers at all expertise levels.

## Graphical Abstract

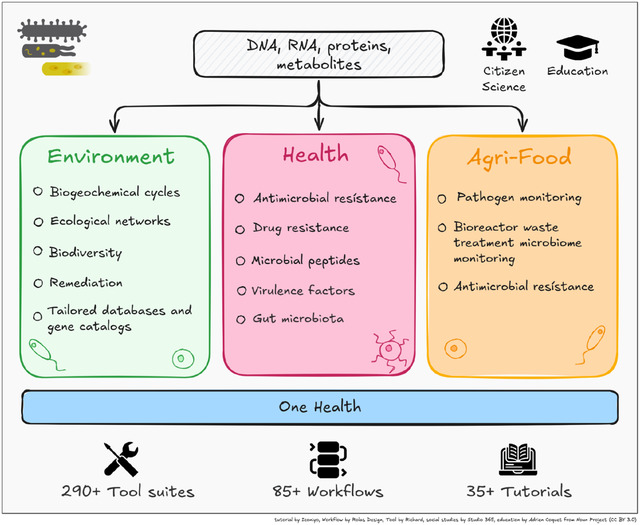

Microbiology has experienced a transformative shift over the past two decades with advances in molecular biology and high-throughput sequencing technologies^1,2^. These innovations have greatly enhanced our ability to characterize microbial composition and function across clinical and environmental contexts. However, the massive volume and diversity of data present major challenges in processing, interpreting, and deriving meaningful insights. Addressing these challenges requires bioinformatics solutions that are robust, accurate, reproducible, and aligned with FAIR^3^ (Findable, Accessible, Interoperable, Reusable) principles.

Effectively analyzing microbial data requires integrating multiple generation methods (e.g. short-read sequencing, mass spectrometry) and analytical approaches, including (meta)genomics, (meta)transcriptomics, and (meta)proteomics. This demands expertise, computational resources, and access to large databases. Choosing appropriate bioinformatics tools and reference databases adds complexity and risks introducing biases. Moreover, global disparities in computational infrastructure limit researchers’ ability to perform large-scale analyses, highlighting the need for an open, standardized, and freely accessible computing environment.

The growing emphasis on FAIR^3^ principles and open practices promotes transparent and reproducible workflows but adds complexity to data organization, annotation, and tool compatibility. Nevertheless, these approaches are particularly valuable in interdisciplinary fields such as One Health^4^, where microbiology intersects with public health, agriculture, and environmental science, fostering collaboration and innovation.

To address the need for flexible and accessible bioinformatics tools and workflows, several platforms have been developed (e.g. MGnify^5^, MG-RAST^6^, bioBakery^7^, Seqera Tower, Epi2Me Desktop, SILVAngs, Anvi’o^8^, KBase^9^; Extended Data Table 1). While these platforms excel in specific domains, many are limited to certain ‘omics modalities, lack interoperability, or function as “black boxes”, reducing user transparency and control. Additionally, limited computational expertise remains a barrier to widespread adoption, underscoring the need for open, integrated, and user-friendly solutions.

Here, we present microGalaxy’s community efforts to build an open-source Galaxy-based platform for microbial data analysis. Galaxy is an open, web-based platform for FAIR-compliant, reproducible research^10^. Since 2016, the Galaxy Project has published biennial papers that are recommended as primary references (https://galaxyproject.org/citing-galaxy/#primary-publication). These publications, including previous core works, have been cited 12,736 times, with 26.78% related to microbiology (Extended Data Fig. 1A). Bacteria, pathogens, and microbiomes are the most studied subjects, with (meta)genomics being the key methodologies and with community profiling and functional analysis studies being the key technical targets (Extended Data Fig. 1B–C).

A 2023 survey (Supplementary Document 1) of the microbial research community highlights Galaxy’s impact and areas for growth (Supplementary Table 1). Consistent with Galaxy citations (Extended Data Fig. 1A), survey results show a focus on bacteria, with metagenomics and single-organism genomics being the most used techniques, particularly for functional analysis, gene identification, and assembly (Extended Data Fig. 2, Supplementary Table 1). Participants identified two major obstacles to using Galaxy effectively: lack of experience and technical challenges. These challenges are being actively addressed through ongoing updates and expanded training resources. The survey also revealed an underrepresentation of researchers from South Asia, South America, and Africa – regions microGalaxy community aims to support by providing free computational resources. Notably, most participants indicated that they would adopt microbial tools if these were available on Galaxy, underscoring the importance of microGalaxy’s role in expanding access to microbial tools.

The Microbiology Galaxy Lab builds on Galaxy’s foundation to address the microbial bioinformatics challenges by offering: (i) access to a diverse range of critically reviewed software tools and workflows, (ii) a user-friendly interface and workflow editor, (iii) free access to public computational resources, (iv) integration with training resources to support users with varying bioinformatics expertise, (v) an Application Programming Interface (API) for automation, integration, and advanced data analysis. By leveraging these features, the Microbiology Galaxy Lab provides a comprehensive framework that supports the global microbiology community in overcoming data and computational barriers.

## Results

### A Microbiology-Focused Galaxy Lab for FAIR and Reproducible Analyses

The Microbiology Galaxy Lab is built on the Galaxy framework^10^, which provides a user-friendly interface for data analysis without requiring programming expertise. Users can run individual tools or create complex workflows, with full provenance tracking to ensure reproducibility, designed specifically for microbiology research. The Microbiology Galaxy Lab offers a tailored collection of tools, workflows, and resources. Available on multiple public Galaxy servers (microbiology.usegalaxy.eu, microbiology.usegalaxy.org, microbiology.usegalaxy.org.au, microbiology.usegalaxy.fr), it provides free access to storage and computational resources up to server-specific limits, enabling users worldwide to perform sophisticated analyses regardless of local infrastructure. Users can upload data locally, via HTTP bulk transfer or commercial clouds, or fetch it automatically from sources like UCSC Genome Browser database^11^, NCBI Sequence Read Archive (SRA)^12^, EMBL-EBI European Nucleotide Archive (ENA)^13^, and MGnify^5^.

### Community-curated tools for microbiology research

**Fig 1:**
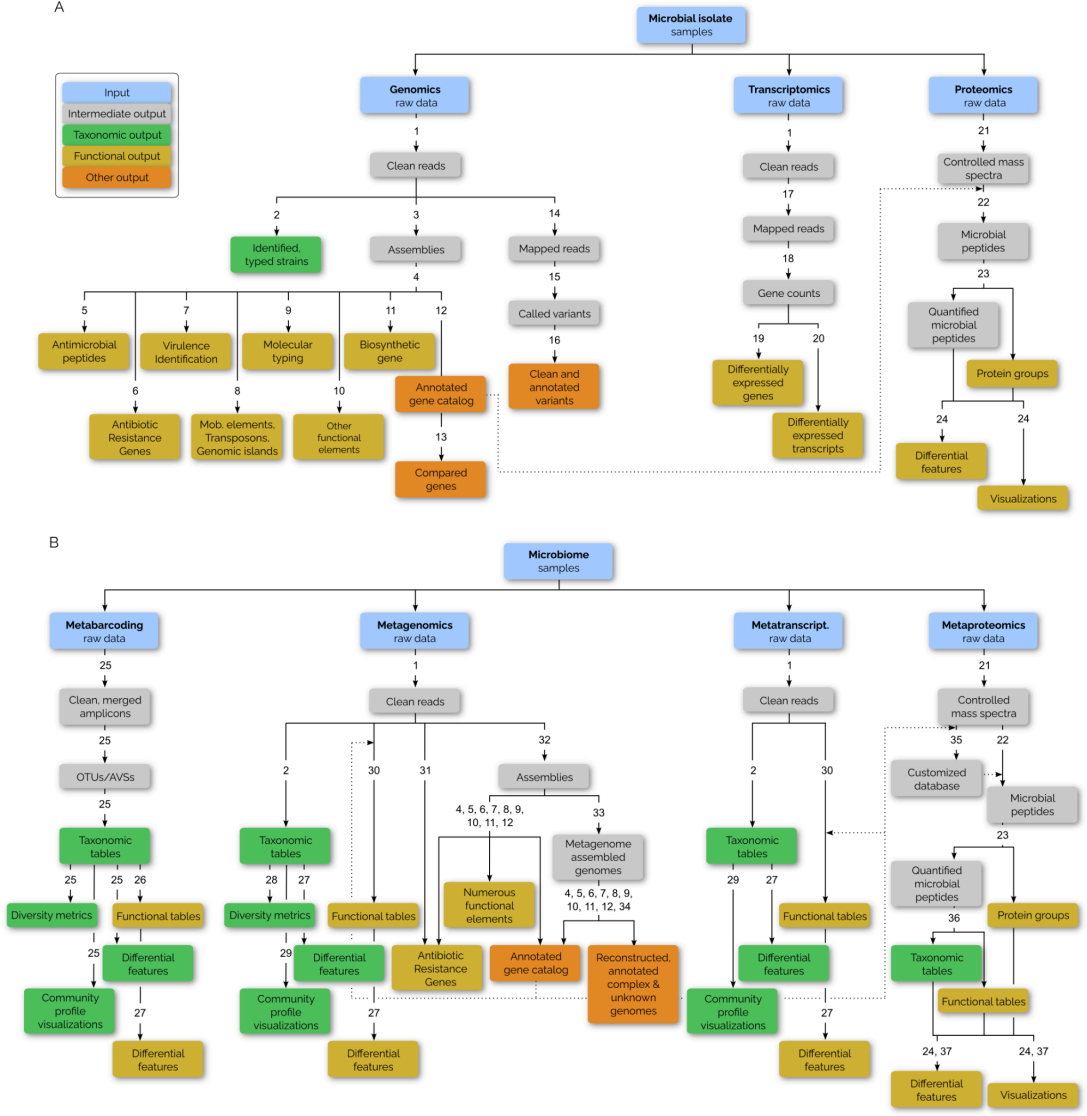
Overview of microbial data analysis tasks with corresponding tool suites and potential analysis available on the Microbiology Galaxy Lab. Analysis tasks, categorized by data type, for (A) microbial isolate samples, including genomics, transcriptomics, and proteomics, and (B) microbiome samples, encompassing metabarcoding, metagenomics, metatranscriptomics, and metaproteomics. Each task progresses from raw data to intermediate and final outputs, with outputs color-coded by type and annotations indicating the tool suites that can be used for these analyses. The legend indicates input data (blue), intermediate outputs (gray), taxonomy related outputs (green), function related outputs (yellow), and other outputs (orange). Numbers in the figure correspond to tool suites: (1) falco (biotools:falco), FastQC (RRID:SCR_014583), Cutadapt (RRID:SCR_011841), fastp (RRID:SCR_016962), MultiQC (RRID:SCR_014982), Porechop (RRID:SCR_016967), Filtlong (RRID:SCR_024020), Nanoplot (RRID:SCR_024128), Pycoqc (RRID:SCR_024185), FastQE (bio.tools:fastqe), lighter (RRID:SCR_024095), Rasusa (biotools:rasusa), BBtools (biotools:bbtools), SeqKit (RRID:SCR_018926), NCBI FCS GX (RRID:SCR_026367), Bowtie (RRID:SCR_005476), BWA (RRID:SCR_010910), minimap (RRID:SCR_018550), SortMeRNA (RRID:SCR_014402); (2) MetaPhlAn (RRID:SCR_004915), Kraken(2) (RRID:SCR_008140), Bracken (bio.tools:bracken), Diamond (RRID:SCR_016071), Sylph (bio.tools:sylph), mOTUs (bio.tools:motus); (Re)Centrifuge (biotools:Recentrifuge), GTDB-Tk (RRID:SCR_019136), VirAnnot (biotools:virannot), Metabuli, MEGAN; (3) Shovill (RRID:SCR_017077), Unicycler (RRID:SCR_024380), SPades (RRID:SCR_000131), Flye (RRID:SCR_017016), SKESA (RRID:SCR_024341), ABySS (RRID:SCR_010709), Velvet (RRID:SCR_010755), Unicycler (RRID:SCR_024380), Racon (RRID:SCR_017642), Pilon (RRID:SCR_014731), Polypolish (biotools:polypolish), QUAST (RRID:SCR_001228), BUSCO (RRID:SCR_015008), CheckM (RRID:SCR_016646); (4) Bakta (RRID:SCR_026400), Prokka (RRID:SCR_014732), Prodigal (RRID:SCR_011936), Maker (RRID:SCR_005309), braker (RRID:SCR_018964), eggNOG Mapper (RRID:SCR_021165), InterProScan (RRID:SCR_005829), Funannotate (RRID:SCR_023039), pharokka (RRID:SCR_026017); (5) HMMER (RRID:SCR_005305); (6) Bakta (RRID:SCR_026400), AMRFinderPlus (biotools:amrfinderplus), ABRicate (RRID:SCR_021093), fARGene (biotools:fargene), hAMRonization (biotools:hamronization), argNorm (biotools:argnorm), StarAMR (biotools:staramr), TB-Profiler (biotools:tb-profiler), SeqSero2 (biotools:SeqSero2), abriTAMR (biotools:abritamr); (7) ABRicate (RRID:SCR_021093); (8) ISEScan (biotools:isescan), Transit (RRID:SCR_016492), PlasmidFinder (biotools:PlasmidFinder); (9) StarAMR (biotools:staramr), chewBBaca (biotools:chewbbaca), pyMLST (biotools:pymlst), MLST (RRID:SCR_010245), SISTR (RRID:SCR_024342); (10) Integron Finder (biotools:integron_finder), CRISPRCasFinder (biotools:CRISPRCasFinder), MITOS (biotools:mitos); (11) antiSMASH (RRID:SCR_022060), HMMER (RRID:SCR_005305); (12) Gene Ontology (RRID:SCR_002811), KEGG (RRID:SCR_012773); (13) Orthofinder (RRID:SCR_017118), Proteinortho, Roary (RRID:SCR_018172), Mauve (RRID:SCR_012852), Clustal (Omega) (RRID:SCR_001591), FastTree (RRID:SCR_015501), RAxML (RRID:SCR_006086), Circos (RRID:SCR_011798), PPanGGOLiN (biotools:PPanGGOLiN), Panaroo (RRID:SCR_021090); (14) BWA (RRID:SCR_010910), Bowtie (RRID:SCR_005476), minimap (RRID:SCR_018550), Picard Tools (RRID:SCR_006525); (15) FreeBayes (RRID:SCR_010761), GATK (RRID:SCR_001876), Bcftools (RRID:SCR_005227), Snippy (RRID:SCR_023572), medaka (biotools:medaka), Clair3 (RRID:SCR_026063); (16) VCFtools (RRID:SCR_001235), SnpSift (RRID:SCR_015624), SnpEff (RRID:SCR_005191), Samtools (RRID:SCR_002105), (17) STAR (RRID:SCR_004463), HISAT (RRID:SCR_015530), Bowtie2 (RRID:SCR_016368); (18) featureCounts (RRID:SCR_012919), HTSeq (RRID:SCR_005514); (19) edgeR (RRID:SCR_012802), DESeq2 (RRID:SCR_015687); (20) StringTie (RRID:SCR_016323), GFFCompare (biotools:gffcompare), Cufflinks (RRID:SCR_014597); (21) msconvert (biotools:msconvert), OpenMS (RRID:SCR_012042); (22) SearchGUI (RRID:SCR_012054), PeptideShaker (RRID:SCR_002520), MaxQuant (RRID:SCR_014485), FragPipe (RRID:SCR_022864); (23) MaxQuant (RRID:SCR_014485), PepQuery (biotools:PepQuery), FragPipe (RRID:SCR_022864), MSstatsTMT (biotools:msstatstmt), iTRAQ (biotools:itraq), UniPept (RRID:SCR_024987); (24) MSStats (RRID:SCR_014353); (25) QIIME2 (RRID:SCR_008249), dada2 (RRID:SCR_023519), LotuS (biotools:LOTUS), mothur (RRID:SCR_011947), mOTUs (bio.tools:motus), FROGs (biotools:frogs); (26) Picrust (RRID:SCR_016855); (27) MaAsLin2 (RRID:SCR_023241), edgeR (RRID:SCR_012802), ANCOM (RRID:SCR_024901), ALDEx2 (RRID:SCR_003364); (28) Krakentools (biotools:krakentools); (29) KRONA (RRID:SCR_012785), GraPhlAn (RRID:SCR_016130), Pavian (RRID:SCR_016679), Phinch (biotools:phinch); (30) HUMAnN (RRID:SCR_014620), minimap (RRID:SCR_018550), BWA-MEM (RRID:SCR_022192), Diamond (RRID:SCR_016071); (31) Groot (biotools:groot), deepARG (biotools:deeparg); (32) MEGAHIT (RRID:SCR_018551), MetaSPades (biotools:metaspades), Flye (RRID:SCR_017016), Unicycler (RRID:SCR_024380), Racon (RRID:SCR_017642), Pilon (RRID:SCR_014731), Polypolish (biotools:polypolish), (meta)QUAST (RRID:SCR_001228); (33) MetaBAT (RRID:SCR_019134), CONCOCT (biotools:concoct), MAxBin (biotools:maxbin), SemiBin (biotools:semibin), DASTool (biotools:dastool), MetaWRAP (biotools:metawrap), dRep (biotools:drep), CoverM (biotools:coverm), CheckM (RRID:SCR_016646), Binette (biotools:binette); (34) GTDB-Tk (RRID:SCR_019136), Contig Annotation Tool (CAT) (RRID:SCR_008421); (35) Metanovo (biotools:metanovo); (36) Unipept (RRID:SCR_024987); (37) MetaQuantome (biotools:metaQuantome).

Among the 10,000 scientific tools^10^ grouped in 1,500 tool suites in the Galaxy ecosystem, the microGalaxy community curates 870 tools across 294 tool suites tailored for microbial research (Fig. 1, Supplementary Table 2). This collection continues to grow (Extended Data Fig. 3C) and has been used over 21 million times in the past five years, highlighting its wide adoption (Extended Data Fig. 3, Supplementary Table 2).

These tools support a broad range of bioinformatics tasks, from genome assembly and taxonomic classification to variant calling and metaproteomics (Fig. 1, Extended Data Fig. 3B–C). Widely used tools include sequence pre-processing (e.g. fastp^14^, Cutadapt^15^), genome annotation (e.g. Bakta^16^), genome assembly (e.g. (meta)SPAdes^17^), and taxonomic classification (e.g. QIIME2^18^, Kraken2^19^), while more specialized tools for microbial ecology and phylogenetics see focused application (Extended Data Fig. 3B–C). This balance ensures the Microbiology Galaxy Lab meets both routine and advanced research needs. The tools are available in the Microbiology Galaxy Lab servers and other public Galaxy instances, such as GalaxyTrakr^20^ and Galaxy@Pasteur (Extended Data Fig. 3A). Researchers can also integrate them into local or cloud-hosted Galaxy setups.

To support these tools, Galaxy hosts extensive reference databases, essential for tasks such as functional annotations and taxonomic classification, distributed via CernVM-FS^21^, a network file system optimized for delivering and distributing scientific software and datasets to computing resources in large-scale distributed computing environments. For example, usegalaxy.eu offers over 170 integrated reference genomes and 11 terabytes of reference data.

Another standout feature of Galaxy is its support for interactive tools, allowing live data exploration within the platform. In addition to general-purpose tools like Jupyter Notebooks (RRID:SCR_018315) and RStudio (RRID:SCR_000432), microbial research tools like Pavian^22^, Phinch^23^, Shiny Phyloseq^24^, and Apollo^25^ enhance user interaction and analysis capabilities.

### Integrated Workflows for Scalable Microbial Analyses

Workflows streamline complex analyses by connecting multiple tools in a reproducible manner. Galaxy provides a robust workflow management system, similar to Nextflow^26^ and Snakemake^27^, but with the advantage of a graphical interface. Users can build pipelines interactively or extract workflows from manual analyses.

The microGalaxy community offers 89 curated, ready-to-use workflows for microbial research (Supplementary Table 3, Extended Data Fig. 4). Forty-two of these workflows have been published on workflow repositories such as WorkflowHub^28^ and Dockstore^29^ and can be executed directly on the Microbiology Galaxy Lab or installed on any Galaxy server. The other workflows are shared across UseGalaxy servers under the *microgalaxy* tag, allowing direct execution or transfer between servers.

Specialized workflows support diverse microbial analyses. For example, ABRomics (https://www.abromics.fr/), a French platform for antibiotic resistance research and surveillance, developed together with the microGalaxy community several workflows for essential processes: quality assessments and read cleaning, taxonomic assignment, genome assembly, and genome annotation steps to identify genes, plasmids, integrons, insertion sequence (IS) elements, and antimicrobial resistance genes. Similarly, PathoGFAIR^30^ workflows facilitate pathogen detection and tracking from metagenomic Nanopore, long-read sequencing, integrating quality control and contamination filtering, taxonomic profiling, phylogenetic identification, and variant calling.

Beyond genomics, metaproteomics workflows cover all analysis steps, from database construction to taxonomic and functional annotation using tools like SearchGUI^31^, PeptideShaker^32^, and Unipept^33^. Multi-omics workflows combine metagenomic assembly, binning, taxonomic classification, and functional annotations with quantitative metatranscriptomics and metaproteomics to facilitate the functional analysis of individual species within a complex microbial ecosystem^34^. In addition, the Galaxy ecosystem also supports metabolomics analysis^35–37^ using both mass spectrometry and NMR data complementing the microbiome-focused multi-omic workflows previously described.

microGalaxy workflows also enhance existing pipelines. In collaboration with EMBL-EBI, the MGnify^5^ amplicon analysis pipeline has been adapted into a customizable Galaxy workflow (https://galaxyproject.org/news/2024-11-15-mgnify-v5/), adding all the previously stated advantages of Galaxy to this workflow

### Resources Backed by Comprehensive Training Support

Training empowers users to execute complex analyses, understand methodologies, and enhance data interpretation. microGalaxy provides extensive training support for tools and workflows through 36 tutorials, 17 videos (16 hours), and 4 structured learning pathways for microbial analyses hosted on the Galaxy Training Network (GTN)^38,39^ (Extended Data Fig. 5, Supplementary Table 4). These resources cover various topics, from basic sequence analysis to advanced metaproteomics or metagenomics assembly (Extended Data Fig. 5), with regular updates ensuring alignment with the latest tools and methodologies. Integrating these materials into the Microbiology Galaxy Lab enhances accessibility and user confidence to undertake complex analyses.

Beyond self-guided resources, training events are critical in skill development. Over the past five years, the microGalaxy community has conducted 30+ training events (Supplementary Table 5), often supported by the Galaxy Training Infrastructure as a Service (TIaaS) framework^40^, which provides dedicated computing resources for efficient data processing during training sessions. A notable example is the 2021 *Analysis of Functions Expressed by Microbiomes* workshop (https://galaxyproject.org/events/2021-11-microbiomes/home/), co-hosted by CSIR-IMTech (Chandigarh, India) and Galaxy-P team (MN, USA), which trained 37 participants in microbiome functional analysis while encouraging collaboration and knowledge sharing among researchers exploring the ecological roles of microbial communities.

The microGalaxy community also contributes to large-scale global initiatives like the Galaxy Training Academy (formerly Galaxy Smörgåsbord). Since 2021, this annual event has attracted over 10,000 participants, offering remote, asynchronous, video-based training with community support on Slack. In 2024, the microGalaxy community offered two dedicated tracks, *Microbiome* and *Bacterial Genomics*, where 66.69% of 3,000+ registrants selected at least one of these tracks. The same model has been used for specialized trainings, such as *Mycobacterium tuberculosis* genomic analysis (https://training.galaxyproject.org/training-material/events/2024-06-10-mtb-ngs.html), reaching broad participation from countries in the global south, where tuberculosis is a major health concern.

### Robust Support and Community Engagement

Beyond training, Microbiology Galaxy Lab users benefit from strong support via the Galaxy Help forum (https://help.galaxyproject.org/) and community-driven platforms like Matrix and Slack. The microGalaxy community, launched in 2021, includes 55+ mailing list members and 75+ Matrix participants, fostering a collaborative atmosphere through regular meetings, working groups, and events. Hackathons, workshops, and meetings drive the development of new workflows, tool improvements, and knowledge sharing. The microGalaxy community has led two ELIXIR BioHackathon projects^41^ and a microGalaxy hackathon^42^ to build the Galaxy Codex, a repository for Galaxy tool, workflow, and training metadata^43^. These efforts enhanced tool and tutorial annotations using the EDAM Ontology^44^ (see Methods). A recent hackathon integrating workflows into the Intergalactic Workflow Commission (IWC) (https://github.com/galaxyproject/iwc) further expanded platform capabilities.

## Discussion

The microGalaxy community, through the Microbiology Galaxy Lab, provides a comprehensive platform with 290+ tools, 85+ workflows, public and locally installed reference databases, 35+ tutorials, and 15+ videos for microbial research. As an open-source, user-friendly solution within the Galaxy ecosystem, it offers extensive capabilities in microbial data analysis. Its workflow manager, user-friendly design, diverse analytical tools (Extended Data Table 1), and powerful computational resources enable researchers to perform complex analyses beyond personal hardware limitations.

### Exemplary Use Cases Empowered by microGalaxy Resources

The microGalaxy community’s resources have been widely applied in microbial research, as evidenced by numerous citations (Extended Data Fig. 1). Below, we highlight key research applications across different fields, with additional use cases in Supplementary Table 6.

First, Galaxy resources are extensively used in microbial genomics for clinical studies. Merdan et al. 2022^45^ identified drug-resistance mutations in *Candida glabrata*, discovering that the 941delC mutation in *ERG1* gene disrupts the ergosterol synthesis pathway, conferring resistance to azole and amphotericin B. In antimicrobial resistance (AMR) detection, Galaxy-based BenchAMRking revealed inconsistencies in AMR gene predictions, leading to improvements in standardization^46^. This work continues with Galaxy@Sciensano (https://galaxy.sciensano.be/), which provides 50+ custom tools for genomic surveillance of pathogens^47^. Clinical metaproteomics holds immense potential in microbiome research for understanding host-microbe interactions underlying human disease. However, challenges persist, primarily in characterizing microbial proteins present in low abundance relative to host proteins. Researchers also face hurdles from using very large protein sequence databases in peptide and protein identification from mass spectrometry (MS) data, as well as performing rigorous analyses of identified peptides, including taxonomic and functional assignments and statistical analysis to determine differentially abundant peptides. Galaxy workflows tackle these challenges, facilitating studies on COVID-19, cystic fibrosis, and ovarian cancer biomarker discovery^48–50^. Applying machine learning (ML) and artificial intelligence (AI) to microbiome research is an area of growing interest, and it has been successfully integrated into Galaxy, allowing researchers to perform more efficient analyses. Cumbo et al. 2024^51^ used AI-driven tools to identify potential microbial biomarkers for colorectal cancer by analyzing public metagenomic samples from patients in a case/control scenario.

In an environmental context, non-targeted studies are challenging and require powerful and reliable bioinformatic tools. Galaxy enables multi-omics studies of microbial ecology. Péguilhan et al. 2023^52^ examined the functioning of microorganisms in the high atmosphere and clouds using combined metagenomics/metatranscriptomics, yielding unprecedented insights. To overcome the high multifactorial variability of microbial assemblages in these environments, a composite reference metagenome was built by creating a catalog of non-redundant genes predicted from all metagenomics sequences and annotated using large functional and taxonomic public databases. This catalog was used as a reference for the differential gene expression analysis from metatranscriptomes. The gene ontology terms (GO) and enzyme commission numbers (E.C.) associated with differentially expressed genes provided consistent pictures of microbial functioning in such unexplored environments. This revealed that multiple biological processes occur in airborne microorganisms, including central metabolic functions and stress responses, and that the presence of condensed water in clouds triggers energy production, fungal spore germination, starvation, and autophagy. Metaproteomics workflows have also been applied to soil and ocean microbiomes. A recent study of North Atlantic Ocean samples^53^ compared workflows for the taxonomic and functional analyses of mass spectrometry data using tools like MaxQuant (v.1.6.17.0)^54^ and SearchGUI (v.3.3.10.1). The results underscored the importance of robust and reproducible workflows in environmental research. Another strategy was used in Schiml et al. 2023^34^ where metaproteomics was combined with metagenomics and metatranscriptomics to study a cellulose-degrading microbial consortium from a biogas reactor in Norway. They employed metagenomics to recover metagenome-assembled genomes (MAGs), including *Hungateiclostridium thermocellum, Thermoclostridium stercorarium*, and multiple heterogenic strains affiliated with *Coprothermobacter proteolyticus*. The predicted genes from the metagenomes have been used to construct databases for mRNA and protein identification and quantification. Metatranscriptomics reveals the functional potential of these microbes, while metaproteomics identifies expressed proteins and active metabolic pathways, linking them to specific MAGs. The integration of these meta-omics techniques within interlaced workflows in Galaxy provided a comprehensive view of microbial interactions and their roles in biomass degradation, with annotations for carbohydrate-active enzymes (CAZymes) and KEGG pathways.

In addition to its applications in research, Galaxy supports participatory science and education. The BeerDEcoded project, led by the Street Science Community (https://streetscience.community/), engages participants from diverse backgrounds to analyze beer samples’ microbiomes. Galaxy’s user-friendly interface and workflows have enabled high school pupils to explore microbiome analysis while learning the principles of open science. Similarly, the BioDIGS project, part of the Genomic Data Science Community Network (GDSCN)^55^, empowers scientists from underserved institutions across the United States to investigate the microbial life of their local environments (https://biodigs.org/). Additionally, the SPUN non-profit organization (https://www.spun.earth/) leverages Galaxy to map mycorrhizal fungal communities globally. These diverse use cases highlight the Microbiology Galaxy Lab’s role in advancing microbial research, fostering collaboration, and making complex analyses accessible to researchers and citizen scientists.

### Future vision of the microGalaxy community

As microbial research evolves, so must the resources for data analysis. The microGalaxy community is committed to adapting alongside these developments by expanding the platform’s capabilities, improving multi-omics integration, and addressing emerging challenges.

As more microbial data is generated, managing computational resources to process it properly and efficiently remains a critical challenge. Tools that predict computational resources based on input dataset requirements will be essential in addressing this challenge. By allowing Galaxy administrators to dynamically allocate the computational needs of the tools with the Total Perspective Vortex (https://github.com/galaxyproject/total-perspective-vortex), the Microbiology Galaxy Lab can ensure that researchers can run large-scale analyses on available infrastructure without overloading resources. This feature will improve everyone’s fair access to Galaxy and high-performance computing resources in microbial research.

Another key focus is enhancing collaboration, exemplified by the integration of QIIME^18^, which benefits from Galaxy’s intuitive tool interface, workflow manager, and public resource servers. Integrating other multifunctional tools like Anvi’o^8^ for microbial genome visualization and Nextstrain^56^ for phylogenomics will enhance in-depth microbial community analysis. Federated data analysis is another priority possible only with collaborations, e.g. with EMBL-EBI and the MGnify team. Integrating the MGnify amplicon pipeline in Galaxy has allowed for scaling of the compute capabilities through public Galaxy servers, combining results from Galaxy analyses and precomputed data from the MGnify database. Like ABRomics, microGalaxy workflows are being incorporated into Bioinformatics Resource Centers (BRCs) like BRC Analytics (https://brc-analytics.org/), enabling seamless multi-omic analyses of infectious disease organisms within a well-established bioinformatics framework.

While Galaxy supports SARS-Cov-2^57^ and MPOX analyses, expanding capabilities for viruses, archaea, and eukaryotes will fill critical gaps in microbial research. These entities, often underrepresented due to the domination of prokaryotic signals^58^, play vital roles in ecosystems like extreme environments or host-associated microbiomes. Furthermore, strengthening support for multi-omics, particularly through holo-omics approaches for host-microbiome integration, will enable a more comprehensive understanding of host-microbiome interactions through (meta)genomics, (meta)transcriptomics, (meta)proteomics, and (meta)metabolomics integration.

As illustrated by the several use cases (Supplementary Table 6), the Microbiology Galaxy Lab can significantly contribute to One Health initiatives by supporting studies on antimicrobial resistance and zoonotic pathogens. Additionally, biodiversity research—especially in monitoring microbial communities in soils, oceans, and other ecosystems—can benefit from integrating microbial and ecological data analyses. Combining microGalaxy with Galaxy-Ecology, which provides a guidance framework for best practices in ecological data analysis^59^, will promote strong interdisciplinary research. This integration will help researchers apply standardized and reproducible approaches to linking microbial insights with ecological ones. As a result, it will enhance our ability to study microbial diversity in response to global changes, improving our understanding of ecosystem health and resilience.

In summary, the Microbiology Galaxy Lab is more than a tool set. It is a dynamic, community-driven infrastructure fostering collaboration, reproducibility, and accessibility. By integrating diverse analytical workflows and prioritizing scalability, the microGalaxy community is uniquely positioned to advance the microbial data analysis field and deepen our understanding of microbial life.

## Methods

### Galaxy framework and the Microbiology Galaxy Lab

The Galaxy framework^10^ is a robust and widely adopted platform enabling accessible and reproducible bioinformatics analyses. The microGalaxy community extends the Galaxy framework using a community-specific interface called Galaxy Lab. A Galaxy Lab, built and deployed with the Galaxy Framework and Galaxy Labs Engine (manuscript in preparation, Wendi A Bacon, BB, Ove J. R. Gustafsson, CH, Winnie Mok, Anna Syme, PZ, GRP), provides users with a focused workspace that retains all the benefits of the Galaxy ecosystem, including accessibility, scalability, and reproducibility, but is tailored specifically to the community needs. With templated elements, each server can host a similar version of a Galaxy Lab, centralizing community resources while allowing adaptations such as server-specific branding and support links.

Focused on microbial research, the Microbiology Galaxy Lab facilitates access to a comprehensive set of tool suites, workflows, and training materials; it ensures that everyone at any level of expertise can efficiently perform analyses, thus fostering a supportive and inclusive environment for microbial research. The Microbiology Galaxy Lab is deployed on public Galaxy servers (usegalaxy.eu, usegalaxy.org, usegalaxy.org.au, usegalaxy.fr), further enhancing its accessibility and usability. Hosted as a dedicated subdomain on these servers, it ensures streamlined access to its tools and workflows while leveraging the robust computational resources provided by the computing infrastructures hosting these servers. With its availability on several major public Galaxy servers, researchers worldwide can access cutting-edge microbiological analysis capabilities without needing local installations or specialized hardware.

All resources supporting the Microbiology Galaxy Lab, including its customized interface, tools, and workflows, are curated and stored within the Galaxy CoDex GitHub repository (https://github.com/galaxyproject/galaxy_codex). The Galaxy CoDex is a centralized repository that ensures the versioning and documentation of microGalaxy components.

### Community-Curated Tools

The tools supported by the microGalaxy community and the Microbiology Galaxy Lab are sourced from the Galaxy ToolShed^60^ (https://toolshed.g2.bx.psu.edu), an extensive repository hosting Galaxy tool wrappers. These tool wrappers provide the integration layer between a command of external software and the Galaxy platform, defining inputs/outputs (including their formats) and parameters. These tool wrappers are developed and maintained by the Galaxy community and groups such as the Intergalactic Utilities Commission (IUC) (https://galaxyproject.org/iuc/), with their source code managed in GitHub repositories. Planemo^61^, a Software Development Kit (SDK) for Galaxy tools, is instrumental in the development process. It is a command-line utility that assists in creating, testing, and validating Galaxy tool wrappers, promoting consistency and high-quality standards across the platform. Once developed and approved by the community, tools are stored in the Galaxy ToolShed for easy access and installation on any Galaxy server. The Galaxy ToolShed is, in this way, an App Store for the Galaxy community.

Any tool dependencies are resolved using packages or containers available through Bioconda^62^ or Biocontainers^63^. When the dependencies are updated, tools maintained by the IUC or other community tool repositories undergo a semi-automated updating process. Once approved by a community member, the updated tool becomes publicly available on ToolShed and is automatically updated on the major Galaxy servers where they are installed. This process ensures that users have access to the latest versions of tools. At the same time, legacy versions are retained to support reproducibility, enabling researchers to repeat analyses even as tools evolve.

Tools in the Galaxy ToolShed are organized into individual entries (a single command) or grouped into tool suites (a set of software commands). The microGalaxy community hosts a subset of these tool suites ready to be used within the Microbiology Galaxy Lab. This ensures a streamlined user experience, making the tools readily available without requiring additional configuration.

### Workflows

Workflows are at the core of reproducible data analysis. microGalaxy community workflows exist at four levels of development, which provide varying degrees of accessibility, adherence to best practices, and maintenance: (i) Publicly shared workflow: These workflows, tagged with #microGalaxy, require minimal effort to create. They are readily accessible to the research community, available across public Galaxy instances, and offer a starting point for users seeking to conduct microbial data analysis; (ii) Workflows in WorkflowHub^28^: Workflows published in WorkflowHub^28^, a workflow registry, are annotated with information such as creator information and licensing. These workflows may not include test data, but they offer documented and structured analysis workflows for microbial research; (iii) Workflows in WorkflowHub related to tutorials: Tutorials on the Galaxy Training Network are recommended to be supported by workflows. These workflows adhere to Galaxy’s best practices, i.e., including clear annotations, proper input and output parameter definitions, formal documentation (such as creator information and licensing), and test data; (iv) IWC-Validated Workflows on WorkflowHub and Dockstore: A subset of workflows available through WorkflowHub has been further curated through the Intergalactic Workflow Commission (IWC) to adhere to Galaxy’s best practices, including test data, and support state-of-the-art analyses. Stored in the IWC GitHub repository, they are supported by a semi-automated updating system: when a Galaxy tool used in the workflow is updated, a continuous integration pipeline tests the workflow with the updated tool version. Once a community member approves, the updated workflow becomes publicly available on WorkflowHub, Dockstore, and the major Galaxy instances. In addition, Galaxy users can easily import workflows from WorkflowHub or Dockstore directly into their Galaxy server, enabling seamless integration into their analysis pipelines. This process ensures that microGalaxy community workflows remain up-to-date and reproducible, making them an invaluable resource for microbial research.

### Training materials

Training is critical to equipping users with the knowledge needed to fully leverage the platform’s capabilities. The global Galaxy community has developed a comprehensive suite of over 400 tutorials, which are available on the Galaxy Training Network (GTN)^38,39^. A subset of these tutorials, tagged with the term “microGalaxy” (https://training.galaxyproject.org/training-material/search2?query=microgalaxy), is dedicated to microbial data analysis. These tutorials cover various topics, from basic genomics to more complex analyses like metaproteomics (https://gxy.io/GTN:T00221), which allows the user to learn how to match mass spectrometry data to peptide sequences, perform taxonomy and functional analysis, and visualize metaproteomics data. They are designed to support learners of varying expertise levels, from beginners to experienced researchers.

The GTN tutorials are continually updated to incorporate state-of-the-art tools, workflows, and methodologies, ensuring users can access current information. Many tutorials are structured into learning pathways, which provide a step-by-step progression of topics, guiding learners through increasingly complex analyses. These learning pathways are designed to build foundational knowledge while advancing skills for handling cutting-edge microbial data.

In addition to written tutorials, the GTN offers recordings as an interactive and flexible learning method. These videos, stored on YouTube, are accompanied by manually curated captions, making them more accessible to a broader audience, including those who may prefer visual or translated content.

The microGalaxy community also offers a range of training events, such as workshops, hackathons, and online seminars. These events allow users to learn, engage directly with experts, share experiences, and deepen their understanding of the platform’s capabilities.

### Resource aggregation and annotation

Resource annotation and ontologies are essential for ensuring consistency and improving discoverability across the Microbiology Galaxy Lab. To achieve this, Galaxy employs the EDAM ontology^44^, a structured vocabulary for bioinformatics concepts, to categorize tools, workflows, and training materials. This ontology-based approach ensures consistent descriptions across the platform, streamlining resource discovery and enabling users to quickly find resources tailored to their research needs.

All Galaxy resources are aggregated into the Galaxy CoDex (https://github.com/galaxyproject/galaxy_codex), a comprehensive catalog that integrates metadata from the Galaxy ecosystem, bio.tools^64,65^, and WorkflowHub^28^. The CoDex enables the creation of lists and widgets that can be embedded into websites, offering seamless access to up-to-date tools, workflows, and training materials.

During the aggregation process, missing tool annotations were identified; hence, an annotation process was essential for resources lacking EDAM annotations, particularly tools not linked to software registries like bio.tools. The microGalaxy community has extensively worked to improve resource annotations during hackathons^41,42^ and other collaborative activities. As a result, over 300 tool suites have been annotated, and more than 30 tutorials have been enriched with relevant EDAM terms. These efforts have enhanced the categorization and discoverability of resources within the Microbiology Galaxy Lab, making it easier for researchers to locate the appropriate resources for their analyses.

### Survey and use cases

A survey was conducted between March and September 2023 to assess the needs, preferences, and challenges of the microbial research community. The survey aimed to gather insights into how researchers interact with Galaxy for microbial research and identify areas for improvement. The survey was developed by the microGalaxy community and was distributed online via the Galaxy Project website, mailing lists, and social media channels. It consisted of 16 questions (Supplementary Document 1) across several categories, including (i) research focus and community demographics, (ii) tools and workflows, (iii) training and support, and (iv) future developments. The survey included multiple-choice, Likert scale, and open-ended questions to allow the collection of both quantitative and qualitative information.

Participation was voluntary, and the purpose of the study was explained at the top of the survey. A total of 130 researchers (Supplementary Table 1) participated in the survey, representing a diverse range of geographical locations, institutions, and research domains. Quantitative responses were analyzed using descriptive statistics to summarize trends and identify prevalent themes. Qualitative responses to open-ended questions were coded and analyzed thematically to extract insights into specific challenges and recommendations.

Survey participants who indicated willingness to be contacted were sent a follow-up invitation, including a structured document (Supplementary Document 2) to collect detailed information about their use cases. This document included (i) research objectives and questions, (ii) methods employed, including experimental techniques, data generation approaches, and analysis pipelines, and (iii) tools and workflows used within and outside Galaxy. The 21 use cases were collected and anonymized (Supplementary Table 6). The compelling use cases were elaborated upon in the main text of this study, and all authors were invited to contribute to the manuscript preparation.

### Citation Extraction and Annotation

To analyze the impact of Galaxy on microbial research, citations of the major Galaxy papers were extracted via Python (v3.8.19, RRID:SCR_008394) within a Jupyter Notebook (v1.0.0, RRID:SCR_018315). The 8 major publications of the Galaxy Project were extracted from the Galaxy Project’s Google Scholar profile (https://scholar.google.com/citations?hl=en&user=3tSiRGoAAAAJ) using scholarly (v 1.7.11)^66^. These publications and their citations were then retrieved on Semantic Scholar^67^ via its Application Programming Interface (API) using requests (v2.32.3). The collected data included the publication years, titles, and abstracts.

Citations were annotated as microbial-related if their titles or abstracts contained at least one of the 23 predefined keywords relevant to microbial research (“bacteri”, “prokaryot”, “microb”, “pathogen”, “virus”, “phage”, “archae”, “flora”, “microecology”, “microorganism”, “micro-organism”, “microbiome”, “microbiota”, “metabarcod”, “16s”, “16 s”, “18s”, “amplicon”, “metataxonom”, “metagenom”, “metatranscriptom”, “metaproteom”, “multi-locus sequence typing”, “multilocus sequence typing”, “mlst”, “otu”). These keywords were chosen to capture diverse aspects of microbial studies, including terms related to targeted organisms (e.g., bacteria, microbiome) and methodologies (e.g., metagenomics, metaproteomics). Each microbial-related citation was further categorized into three dimensions given keywords in their titles and abstracts to enable a detailed analysis of the research themes addressed in the citing papers: (i) Targeted Organisms (Bacteria, Virus, Archaea, Eukaryote, Microbiome, Pathogen), (ii) Technical Targets (Isolate, Community (taxonomy) profiling, Functional analysis, Interactome, AMR, MAGs, Gene identification / Biomarker, SNP, (M)LST, Annotation, Variant, Comparative analysis), and (iii) Methods (Metabarcoding, (Meta)genomics, Metagenomics, (Meta)transcriptomics, Metatranscriptomics, (Meta)proteomics, Metaproteomics, Metabolomics, Imaging).

### Data visualisation

To analyze and visualize summary data presented in this study, a set of R Markdown (https://github.com/rstudio/rmarkdown) scripts was created and hosted in the GitHub repository dedicated to this article. These scripts streamlined the generation of figures and statistical analyses, allowing for reproducibility and updates as new data become available. Each script ingests tables generated by Galaxy CoDex, filtered for the microGalaxy community, containing metadata and EDAM annotations for tools, training materials, and workflows. This setup enables the calculation of key metrics such as total counts, distribution across EDAM terms, and user feedback statistics.

All analyses were run on R (v4.3.1, RRID:SCR_001905). data.table (v1.14.8, RRID:SCR_026117) and tidyr (v1.1.3, RRID:SCR_017102) libraries were used for data manipulation, with stringr (v1.5.0, RRID:SCR_022813) aiding in text processing. Visualizations were primarily created with ggplot2 (v3.4.4, RRID:SCR_014601), while ggrepel (v0.9.3, RRID:SCR_017393), ggtext (v0.1.2, RRID:SCR_026470), and ggh4x (v0.2.5) were used for labelling, rich text, and facet customization, respectively. Colorspace, and paletteer (v2.1–0) ensured accessible colour schemes, packcircles (v0.3.5) handled circle-packing layouts, and shadowtext (v0.1.2) improved label readability. Additionally, extrafont (v0.18) was used to manage custom fonts.

## Supplementary Material

Supplement 1 **Supplementary Document 1**: **Complete list of survey questions shared with the microbiology research community via a Google Form between March and September 2023**. The survey aimed to assess researchers’ needs, challenges, and preferences in using microbial data analysis tools and platforms.

Supplement 2 **Supplementary Document 2**: **Template to collect detailed use cases from survey participants who expressed interest in contributing to the planned manuscript**. The document includes sections for contact details, research questions, methods, tools, workflows, and results, along with prompts for providing a detailed description of the use cases. Preliminary survey results are also shared to provide context and highlight the motivation for the study.

Supplement 3 **Extended Data Fig. 1: Citation Trends and Microbial Research Topics in Galaxy Publications.** The citations were extracted from the Galaxy Project’s Google Scholar profile, and additional details were retrieved using Semantic Search. (A) Annual publication trends show the total number of citations (blue) alongside those specific to microbial research (brown). (B) Breakdown of microbial-focused citations by Targeted Organisms, Technical Targets, and Methods. Categories were annotated based on predefined keywords found in the title or abstract of each publication

Supplement 4 **Extended Data Fig. 2: Survey results from the microbiology research community (March–September 2023).** Anonymous responses highlighting the main research targets, techniques, and analyses used or desired by microbial researchers. The figure also reports challenges faced by users who rarely or never use Galaxy, survey responses by continent, and preferences regarding tool deployment in Galaxy. Percentages are computed independently for each question based on the total number of responses available for that question. Additionally, some questions, such as “Which analyses do you use or would you like to do?” allowed multiple responses, meaning percentages may sum to more than 100%.

Supplement 5 **Extended Data Fig. 3: Availability, usability, and growth of microbiology-related tool suites within the Galaxy ecosystem.** (A) Heatmap illustrating the availability of microbiology-related tool suites (x-axis) across various Galaxy servers (y-axis), grouped by EDAM topics. Tool suites may appear in multiple groups as they can be annotated with several topics. A logarithmic scale is applied for improved visualization. (B) Scatter plot showing the usage of microbiology-related tool suites over the past five years across all Galaxy main servers, categorized by their EDAM operations. The x-axis represents the total number of suite runs, while the y-axis denotes the total number of tool users. (C) Cumulative number of microbiology-related tools added to Galaxy over time, based on the date of the first commit for each tool suite.

Supplement 6 **Extended Data Fig. 4: Usage of microbiology-related tool suites across workflows.** Heatmap illustrating the presence of microbiology-related tool suites within the available microbiology-related workflows, grouped by four levels of development. The tool suites are organized based on their corresponding EDAM operations, highlighting the breadth of tools utilized for different workflows.

Supplement 7 **Extended Data Fig. 5: Usage of microbiology-related tool suites across training materials.** Heatmap illustrating the presence of microbiology-related tool suites within the available microbiology-related training materials, grouped by EDAM topics. The tool suites are organized based on their corresponding EDAM operations, highlighting the breadth of tools utilized for different training contexts.

Supplement 8 **Supplementary Table 1**: **Anonymous results from the microbiology research community survey conducted between March and September 2023.** The table includes aggregated and anonymized responses to survey questions, detailing researchers’ demographics, tool usage, training needs, and suggestions for future improvements to the Galaxy platform.

Supplement 9 **Supplementary Table 2**: **Overview of the 294 microbiology-related tool suites available.** The table includes detailed metadata for each tool suite, such as suite ID, tool IDs, description, first commit date, homepage, version, conda package details, version status, and ToolShed categories. It also incorporates EDAM annotations (operations, reduced operations, topics, and reduced topics) and links to external resources like bio.tools (ID, name, and description). Usage statistics from Galaxy’s main servers over the last five years, including the number of users and tool runs, are also provided to highlight adoption and activity levels.

Supplement 10 **Supplementary Table 3**: **Comprehensive list of microbiology-related workflows from the microGalaxy community**. This table documents 96 workflows, detailing their names, sources, unique identifiers, and links to relevant repositories. Additional information includes the creators, associated tags, creation and update timestamps, latest and available versions, number of steps, and tools used. The workflows are categorized by EDAM operations and topics, and information on licensing, DOI assignments, and related projects is provided.

Supplement 11 **Supplementary Table 4**: **Comprehensive list of tutorials from the Galaxy Training Network (GTN) specifically dedicated to microbial data analysis**. This table documents 37 tutorials tagged with the term “microGalaxy” (https://training.galaxyproject.org/training-material/search2?query=microgalaxy), providing detailed metadata on each tutorial, including its topic, title, link, EDAM topic and operation annotations, creation and last modification dates, and version history. The table also includes information on available training materials such as slides, videos, workflows, and tool listings. Server support is detailed, distinguishing between precise tool versions and different tool versions. User engagement metrics, including feedback count and mean rating, visitor numbers, page views, visit duration, and video views, highlight the usage and impact of these tutorials.

Supplement 12 **Supplementary Table 5**: **Overview of microbiology-related training events conducted between 2019 and 2024.** The table provides details for 38 events, including their start and end dates, titles, covered topics, target audiences, and the specific contributions of the microGalaxy community when the event encompassed broader subjects beyond microbial data analysis. Links to event pages and the resources utilized during the training sessions are also included.

Supplement 13 **Extended Data Table 1: Features and capabilities of 44 existing user-friendly microbiology data analysis platforms (non-exhaustive list).** The table includes columns detailing general characteristics (e.g., free usage, open source, workflow/pipeline manager), accessibility and availability (e.g., simple end-user modification, user-friendly interface, publicly available web server, automatable API, last update), user support and documentation (e.g., tutorials, documentation, user support), possible methods used to generate input data (e.g., isolate genomics, amplicon/metabarcoding, WGS/metagenomics, metatranscriptomics), and supported analyses (e.g., QC, taxonomy profiling, MAGs, comparative omics, multi-omics integration, interactive visualizations).

Supplement 14 **Supplementary Table 6: Summary of use case submissions collected from survey participants**. The table includes anonymized details of each use case, such as the research question, experimental methods, data analysis approaches, Galaxy tools and workflows used, and specific benefits of using Galaxy. It also highlights the status of the use cases (e.g., starting, ongoing, completed) and any associated publications. This summary serves as a foundation for the Discussion section of the manuscript.

## Data Availability

All supplementary tables associated with this study are available in the dedicated GitHub repository (https://github.com/usegalaxy-eu/microgalaxy_paper_2025). To ensure reproducibility and long-term accessibility, a versioned release of the dataset was created on Zenodo^68^ on March 26th, 2025. Any updates to the repository will be tracked, but the version archived on Zenodo corresponds to the data used in this study.

## References

[1] Di Bella J. M., Bao Y., Gloor G. B., Burton J. P. & Reid G. High throughput sequencing methods and analysis for microbiome research. J. Microbiol. Methods 95, 401–414 (2013). 24029734 10.1016/j.mimet.2013.08.011

[2] Reuter J. A., Spacek D. V. & Snyder M. P. High-Throughput Sequencing Technologies. Mol. Cell 58, 586–597 (2015). 26000844 10.1016/j.molcel.2015.05.004 PMC4494749

[3] Wilkinson M. D. The FAIR Guiding Principles for scientific data management and stewardship. Sci. Data 3, 160018 (2016). 26978244 10.1038/sdata.2016.18 PMC4792175

[4] One Health High-Level Expert Panel (OHHLEP) One Health: A new definition for a sustainable and healthy future. PLOS Pathog. 18, e1010537 (2022). 35737670 10.1371/journal.ppat.1010537 PMC9223325

[5] Richardson L. MGnify: the microbiome sequence data analysis resource in 2023. Nucleic Acids Res. 51, D753–D759 (2023). 36477304 10.1093/nar/gkac1080 PMC9825492

[6] Keegan K. P., Glass E. M. & Meyer F. MG-RAST, a Metagenomics Service for Analysis of Microbial Community Structure and Function. in Microbial Environmental Genomics (MEG) (eds. Martin F. & Uroz S.) vol. 1399 207–233 (Springer New York, New York, NY, 2016). 10.1007/978-1-4939-3369-3_13 26791506

[7] McIver L. J. bioBakery: a meta’omic analysis environment. Bioinformatics 34, 1235–1237 (2018). 29194469 10.1093/bioinformatics/btx754 PMC6030947

[8] Eren A. M. Community-led, integrated, reproducible multi-omics with anvi’o. Nat. Microbiol. 6, 3–6 (2020). 10.1038/s41564-020-00834-3 PMC8116326 33349678

[9] Arkin A. P. KBase: The United States Department of Energy Systems Biology Knowledgebase. Nat. Biotechnol. 36, 566–569 (2018). 29979655 10.1038/nbt.4163 PMC6870991

[10] The Galaxy Community. The Galaxy platform for accessible, reproducible, and collaborative data analyses: 2024 update. Nucleic Acids Res. 52, W83–W94 (2024). 38769056 10.1093/nar/gkae410 PMC11223835

[11] Nassar L. R. The UCSC Genome Browser database: 2023 update. Nucleic Acids Res. 51, D1188–D1195 (2023). 36420891 10.1093/nar/gkac1072 PMC9825520

[12] Sayers E. W. Database resources of the national center for biotechnology information. Nucleic Acids Res. 50, D20–D26 (2022). 34850941 10.1093/nar/gkab1112 PMC8728269

[13] Burgin J. The European Nucleotide Archive in 2022. Nucleic Acids Res. 51, D121–D125 (2023). 36399492 10.1093/nar/gkac1051 PMC9825583

[14] Chen S., Zhou Y., Chen Y. & Gu J. fastp: an ultra-fast all-in-one FASTQ preprocessor. Bioinformatics 34, i884–i890 (2018). 30423086 10.1093/bioinformatics/bty560 PMC6129281

[15] Martin M. Cutadapt removes adapter sequences from high-throughput sequencing reads. EMBnet.journal 17, 10–12 (2011).

[16] Schwengers O. Bakta: rapid and standardized annotation of bacterial genomes via alignment-free sequence identification. Microb. Genomics 7, 000685 (2021). 10.1099/mgen.0.000685 PMC8743544 34739369

[17] Bankevich A. SPAdes: A New Genome Assembly Algorithm and Its Applications to Single-Cell Sequencing. J. Comput. Biol. 19, 455–477 (2012). 22506599 10.1089/cmb.2012.0021 PMC3342519

[18] Bolyen E. Reproducible, interactive, scalable and extensible microbiome data science using QIIME 2. Nat. Biotechnol. 37, 852–857 (2019). 31341288 10.1038/s41587-019-0209-9 PMC7015180

[19] Wood D. E., Lu J. & Langmead B. Improved metagenomic analysis with Kraken 2. Genome Biol. 20, 257 (2019). 31779668 10.1186/s13059-019-1891-0 PMC6883579

[20] Gangiredla J. GalaxyTrakr: a distributed analysis tool for public health whole genome sequence data accessible to non-bioinformaticians. BMC Genomics 22, 114 (2021). 33568057 10.1186/s12864-021-07405-8 PMC7877046

[21] Blomer J., Buncic P. & Fuhrmann T. CernVM-FS: delivering scientific software to globally distributed computing resources. in Proceedings of the first international workshop on Network-aware data management 49–56 (Association for Computing Machinery, New York, NY, USA, 2011). doi:10.1145/2110217.2110225.

[22] Breitwieser F. P. & Salzberg S. L. Pavian: interactive analysis of metagenomics data for microbiome studies and pathogen identification. Bioinformatics 36, 1303–1304 (2020). 31553437 10.1093/bioinformatics/btz715 PMC8215911

[23] Bik H. M. & Inc P. I. Phinch: An interactive, exploratory data visualization framework for –Omic datasets. 009944 Preprint at 10.1101/009944 (2014).

[24] McMurdie P. J. & Holmes S. Shiny-phyloseq: Web application for interactive microbiome analysis with provenance tracking. Bioinformatics 31, 282–283 (2015). 25262154 10.1093/bioinformatics/btu616 PMC4287943

[25] Lewis S. Apollo: a sequence annotation editor. Genome Biol. 3, research0082.1 (2002). 10.1186/gb-2002-3-12-research0082 PMC151184 12537571

[26] Di Tommaso P. Nextflow enables reproducible computational workflows. Nat. Biotechnol. 35, 316–319 (2017). 28398311 10.1038/nbt.3820

[27] Mölder F. Sustainable data analysis with Snakemake. F1000Research 10, 33 (2021). 34035898 10.12688/f1000research.29032.1 PMC8114187

[28] Gustafsson O. J. R. WorkflowHub: a registry for computational workflows. Preprint at 10.48550/ARXIV.2410.06941 (2024). PMC12095652 40399296

[29] Yuen D. The Dockstore: enhancing a community platform for sharing reproducible and accessible computational protocols. Nucleic Acids Res. 49, W624–W632 (2021). 33978761 10.1093/nar/gkab346 PMC8218198

[30] Nasr E., Henger A., Grüning B., Zierep P. & Batut B. PathoGFAIR: a collection of FAIR and adaptable (meta)genomics workflows for (foodborne) pathogens detection and tracking. Preprint at 10.1101/2024.06.26.600753 (2024). PMC12466118 41004266

[31] Barsnes H. & Vaudel M. SearchGUI: A Highly Adaptable Common Interface for Proteomics Search and de Novo Engines. J. Proteome Res. 17, 2552–2555 (2018). 29774740 10.1021/acs.jproteome.8b00175

[32] Vaudel M. PeptideShaker enables reanalysis of MS-derived proteomics data sets. Nat. Biotechnol. 33, 22–24 (2015). 25574629 10.1038/nbt.3109

[33] Vande Moortele T. Unipept in 2024: Expanding Metaproteomics Analysis with Support for Missed Cleavages and Semitryptic and Nontryptic Peptides. J. Proteome Res. acs.jproteome.4c00848 (2025) doi:10.1021/acs.jproteome.4c00848. 39792626

[34] Schiml V. C. Integrative meta-omics in Galaxy and beyond. Environ. Microbiome 18, 56 (2023). 37420292 10.1186/s40793-023-00514-9 PMC10329324

[35] Peters K. PhenoMeNal: processing and analysis of metabolomics data in the cloud. GigaScience 8, giy149 (2019). 30535405 10.1093/gigascience/giy149 PMC6377398

[36] Giacomoni F. Workflow4Metabolomics: a collaborative research infrastructure for computational metabolomics. Bioinforma. Oxf. Engl. 31, 1493–1495 (2015). 10.1093/bioinformatics/btu813 PMC4410648 25527831

[37] Davidson R. L., Weber R. J. M., Liu H., Sharma-Oates A. & Viant M. R. Galaxy-M: a Galaxy workflow for processing and analyzing direct infusion and liquid chromatography mass spectrometry-based metabolomics data. GigaScience 5, 10 (2016). 26913198 10.1186/s13742-016-0115-8 PMC4765054

[38] Batut B. Community-Driven Data Analysis Training for Biology. Cell Syst. 6, 752–758.e1 (2018). 29953864 10.1016/j.cels.2018.05.012 PMC6296361

[39] Hiltemann S. Galaxy Training: A powerful framework for teaching! PLOS Comput. Biol. 19, e1010752 (2023). 36622853 10.1371/journal.pcbi.1010752 PMC9829167

[40] Rasche H. Training Infrastructure as a Service. GigaScience 12, giad048 (2023). 10.1093/gigascience/giad048 PMC10316688 37395629

[41] Zierep P. How to increase the findability, visibility, and impact of Galaxy tools for your scientific community. Preprint at 10.37044/osf.io/qjbxc (2024).

[42] Batut B. How to improve the annotation of Galaxy resources? Outcomes of an online hackathon for improving the annotation of Galaxy resources for microbial data resources. Preprint at 10.37044/osf.io/s7tru (2024).

[43] Batut B. Galaxy CoDex for finding tools, workflows, and training. in F1000Research vol. 13 (2024).

[44] Ison J. EDAM: an ontology of bioinformatics operations, types of data and identifiers, topics and formats. Bioinformatics 29, 1325–1332 (2013). 23479348 10.1093/bioinformatics/btt113 PMC3654706

[45] Merdan O. Investigation of the Defective Growth Pattern and Multidrug Resistance in a Clinical Isolate of Candida glabrata Using Whole-Genome Sequencing and Computational Biology Applications. Microbiol. Spectr. 10, e00776–22 (2022). 35867406 10.1128/spectrum.00776-22 PMC9430859

[46] Strepis N. BenchAMRking: a Galaxy-based platform for illustrating the major issues associated with current antimicrobial resistance (AMR) gene prediction workflows. BMC Genomics 26, 27 (2025). 39794695 10.1186/s12864-024-11158-5 PMC11724594

[47] Bogaerts B. Galaxy @Sciensano: a comprehensive bioinformatics portal for genomics-based microbial typing, characterization, and outbreak detection. BMC Genomics 26, 20 (2025). 39780046 10.1186/s12864-024-11182-5 PMC11715294

[48] Bihani S. Metaproteomic Analysis of Nasopharyngeal Swab Samples to Identify Microbial Peptides in COVID-19 Patients. J. Proteome Res. 22, 2608–2619 (2023). 37450889 10.1021/acs.jproteome.3c00040

[49] Do K. A novel clinical metaproteomics workflow enables bioinformatic analysis of host-microbe dynamics in disease. mSphere 9, e00793–23 (2024). 38780289 10.1128/msphere.00793-23 PMC11332332

[50] Kruk M. E. An integrated metaproteomics workflow for studying host-microbe dynamics in bronchoalveolar lavage samples applied to cystic fibrosis disease. mSystems 9, e00929–23 (2024). 10.1128/msystems.00929-23 PMC11264604 38934598

[51] Cumbo F., Truglia S., Weitschek E. & Blankenberg D. Feature selection with vector-symbolic architectures: a case study on microbial profiles of shotgun metagenomic samples of colorectal cancer. Preprint at 10.1101/2024.11.18.624180 (2024). PMC12018301 40269516

[52] Péguilhan R. Clouds influence the functioning of airborne microorganisms. Biogeosciences 22, 1257–1275 (2025).

[53] Saito M. A. Results from a multi-laboratory ocean metaproteomic intercomparison: effects of LC-MS acquisition and data analysis procedures. Biogeosciences 21, 4889–4908 (2024).

[54] Cox J. & Mann M. MaxQuant enables high peptide identification rates, individualized p.p.b.-range mass accuracies and proteome-wide protein quantification. Nat. Biotechnol. 26, 1367–1372 (2008). 19029910 10.1038/nbt.1511

[55] Network T. G. D. S. C. Diversifying the genomic data science research community. Genome Res. 32, 1231–1241 (2022). 35858750 10.1101/gr.276496.121 PMC9341509

[56] Hadfield J. Nextstrain: real-time tracking of pathogen evolution. Bioinformatics 34, 4121–4123 (2018). 29790939 10.1093/bioinformatics/bty407 PMC6247931

[57] Maier W. Ready-to-use public infrastructure for global SARS-CoV-2 monitoring. Nat. Biotechnol. 39, 1178–1179 (2021). 34588690 10.1038/s41587-021-01069-1 PMC8845060

[58] Bazant W., Blevins A. S., Crouch K. & Beiting D. P. Improved eukaryotic detection compatible with large-scale automated analysis of metagenomes. Microbiome 11, 72 (2023). 37032329 10.1186/s40168-023-01505-1 PMC10084625

[59] Royaux C. Guidance framework to apply best practices in ecological data analysis: lessons learned from building Galaxy-Ecology. GigaScience 14, giae122 (2025). 39937595 10.1093/gigascience/giae122 PMC11816794

[60] Blankenberg D. Dissemination of scientific software with Galaxy ToolShed. Genome Biol. 15, 403 (2014). 25001293 10.1186/gb4161 PMC4038738

[61] Bray S. The Planemo toolkit for developing, deploying, and executing scientific data analyses in Galaxy and beyond. Genome Res. 33, 261–268 (2023). 36828587 10.1101/gr.276963.122 PMC10069471

[62] The Bioconda Team Bioconda: sustainable and comprehensive software distribution for the life sciences. Nat. Methods 15, 475–476 (2018). 29967506 10.1038/s41592-018-0046-7 PMC11070151

[63] Da Veiga Leprevost F. BioContainers: an open-source and community-driven framework for software standardization. Bioinformatics 33, 2580–2582 (2017). 28379341 10.1093/bioinformatics/btx192 PMC5870671

[64] Ison J. Tools and data services registry: a community effort to document bioinformatics resources. Nucleic Acids Res. 44, D38–D47 (2016). 26538599 10.1093/nar/gkv1116 PMC4702812

[65] Ison J. The bio.tools registry of software tools and data resources for the life sciences. Genome Biol. 20, 164 (2019). 31405382 10.1186/s13059-019-1772-6 PMC6691543

[66] Cholewiak S., Ipeirotis P., Silva V. & Kannawadi A. scholarly. Zenodo 10.5281/ZENODO.7542349 (2023).

[67] Kinney R. The Semantic Scholar Open Data Platform. Preprint at 10.48550/ARXIV.2301.10140 (2023).

[68] Nasr E., Pechlivanis N., Zierep P. & Batut B. usegalaxy-eu/microgalaxy_paper_2025: v1.0.0. Zenodo 10.5281/zenodo.15088383 (2025).

