# Supplementary material for: The Microbiology Galaxy Lab: A community-driven gateway to tools, workflows, and training for reproducible and FAIR analysis of microbial data
Source: bioRxiv. 2025 Mar 26:2024.12.23.629682. Originally published 2024 Dec 27. Preprint. [Version 2] doi: 10.1101/2024.12.23.629682 (PMC11703195; doi:10.1101/2024.12.23.629682)

Microbial data analysis in Galaxy

Case studies

*You filled out our form "Evaluation of uses and needs for microbial data analysis in Galaxy". Thanks to you and the community, we collected **more than 100 answers**, which has given us valuable information about the community.*

*In your survey answer, you showed interest in being contacted by the microGalaxy team and contributing an example use case to the planned manuscript. You already gave us some basic information in the form, but **we would appreciate a more detailed description of your work**. We therefore request that you fill out the fields below **before August 31st**, so that we have a good overview of your use case.*

*We plan to **review and group case studies by topics,** and then **highlight especially intriguing ones** (after discussion with the community and you) in the main text of the paper. **All submitted studies (including yours) will be included in the Supplementary material,** and we invite the authors of the selected studies to become **coauthors of the manuscript.***

Thank you for your help!

Contact

First name	Last name	Email	Affiliation	Country	ORCID

Case study

Title

Status

Possible answers: Starting / On-going / Completed / Other

Publications

Funding

Keyword
Give 3-8 keywords describing your use case

Research question

Main target
Keywords. E.g. AMR, Pathogen, Microbiome community, MAGs building, Functional analysis, Gene identification, Comparative analysis, SNP analysis, or anything else

Short description of the research questions of your case study
Images can also be included

Methods

Experimental or Measurement technique used
E.g. metagenomics, metabarcoding, amplicon, proteomics, metabolomics, transcriptomics, single organism genomics

Data generation approach
E.g. Illumina, Nanopore, LS/MS

Data analysis approach
E.g. quality control, taxonomy profiling, assembly, differential gene expression, statistical analysis

Tools, workflows or platforms outside Galaxy

Galaxy tools used

Workflow(s) used

Specific benefits of using Galaxy
E.g. access to computational power, access to databases, tool availability, easy-to-use workflows, reproducibility, bulk processing using collections, or anything else

Short description of the methods you used
Images can also be included

Interesting results

Short description of the results you got
Images can also be included

Other

Anything else you would like to mention about your use case?

References

Teaser of survey's results

If you are interested to have a first peek at the results of the survey, here are some figures based on the first 90 answers.

The figure below shows the main techniques used by the participants.

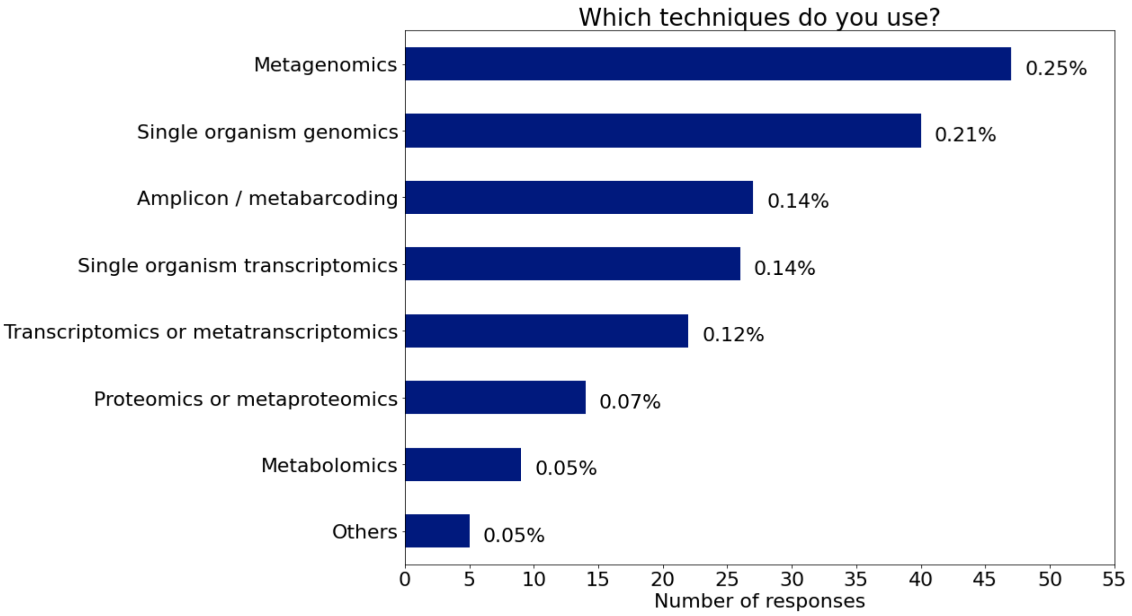

We also discovered that a large majority of the respondents would use Galaxy for their analyses if the features they require were available on the platform. This motivates us to write this paper and take the next steps towards improving Galaxy for the microbial analysis community.

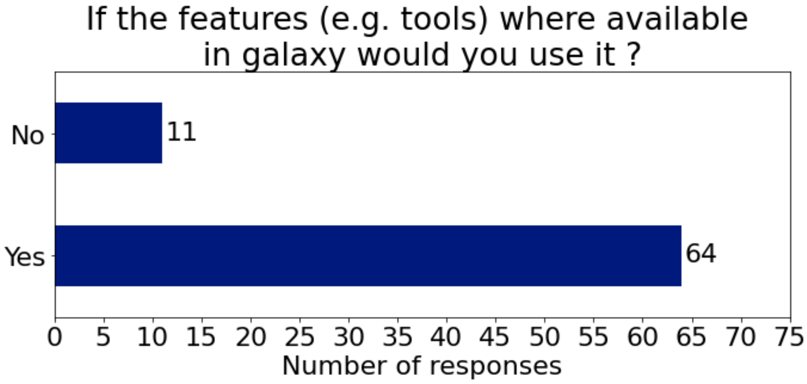

Supplement: Supplementary file 2 [file media-2.pdf]
