# Supplementary material for: The Microbiology Galaxy Lab: A community-driven gateway to tools, workflows, and training for reproducible and FAIR analysis of microbial data
Source: bioRxiv. 2025 Mar 26:2024.12.23.629682. Originally published 2024 Dec 27. Preprint. [Version 2] doi: 10.1101/2024.12.23.629682 (PMC11703195; doi:10.1101/2024.12.23.629682)
Supplement: Supplementary file 3 [file media-3.pdf]

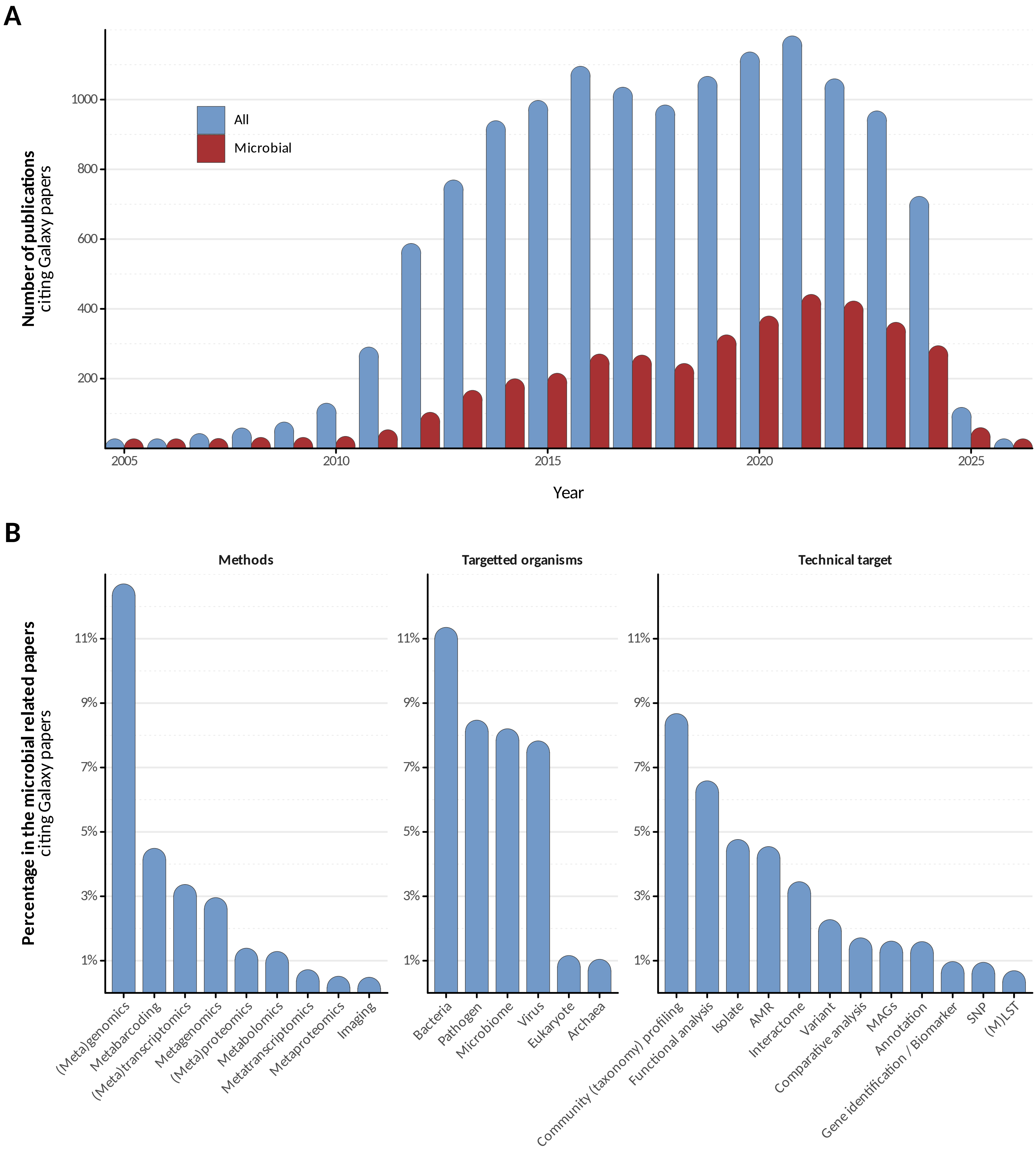

A
Number of publications citing Galaxy papers
All
Microbial
1000
800
600
400
200
2005
2010
2015
2020
2025
Year
B
Methods
Targetted organisms
Technical target
Percentage in the microbial related papers citing Galaxy papers
11%
9%
7%
5%
3%
1%
(Meta)genomics
Metabarcoding
(Meta)transcriptomics
Metagenomics
(Meta)proteomics
Metabolomics
Metatranscriptomics
Metaproteomics
Imaging
Bacteria
Pathogen
Microbiome
Virus
Eukaryote
Archaea
Community (taxonomy) profiling
Functional analysis
Isolate
AMR
Interactome
Variant
Comparative analysis
MAGs
Annotation
Gene identification / Biomarker
SNP
(M)LST
