# Supplementary material for: The Microbiology Galaxy Lab: A community-driven gateway to tools, workflows, and training for reproducible and FAIR analysis of microbial data
Source: bioRxiv. 2025 Mar 26:2024.12.23.629682. Originally published 2024 Dec 27. Preprint. [Version 2] doi: 10.1101/2024.12.23.629682 (PMC11703195; doi:10.1101/2024.12.23.629682)
Supplement: Supplementary file 4 [file media-4.pdf]

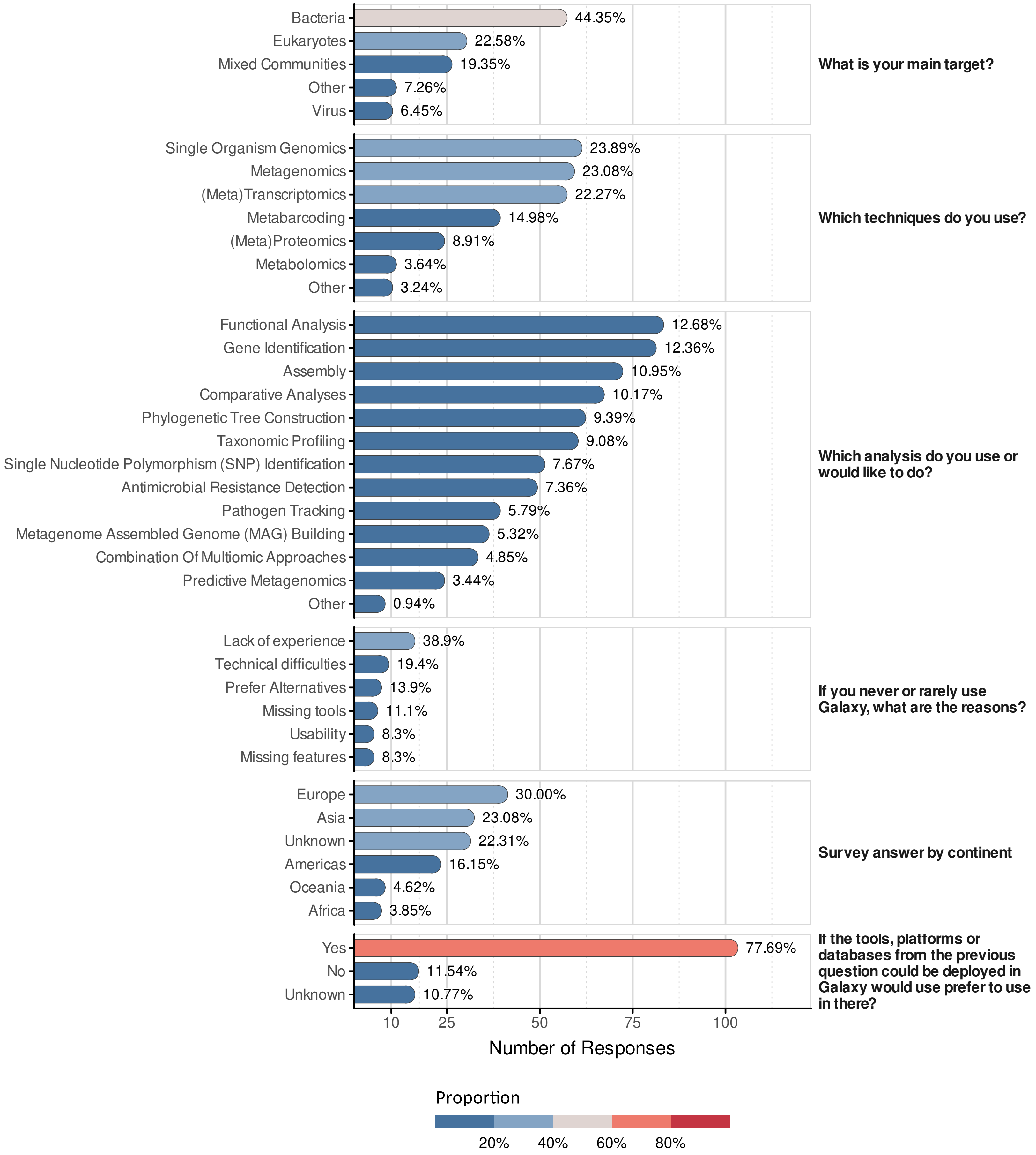
What is your main target?
Bacteria 44.35%
Eukaryotes 22.58%
Mixed Communities 19.35%
Other 7.26%
Virus 6.45%
Which techniques do you use?
Single Organism Genomics 23.89%
Metagenomics 23.08%
(Meta)Transcriptomics 22.27%
Metabarcoding 14.98%
(Meta)Proteomics 8.91%
Metabolomics 3.64%
Other 3.24%
Which analysis do you use or would like to do?
Functional Analysis 12.68%
Gene Identification 12.36%
Assembly 10.95%
Comparative Analyses 10.17%
Phylogenetic Tree Construction 9.39%
Taxonomic Profiling 9.08%
Single Nucleotide Polymorphism (SNP) Identification 7.67%
Antimicrobial Resistance Detection 7.36%
Pathogen Tracking 5.79%
Metagenome Assembled Genome (MAG) Building 5.32%
Combination Of Multiomic Approaches 4.85%
Predictive Metagenomics 3.44%
Other 0.94%
If you never or rarely use Galaxy, what are the reasons?
Lack of experience 38.9%
Technical difficulties 19.4%
Prefer Alternatives 13.9%
Missing tools 11.1%
Usability 8.3%
Missing features 8.3%
Survey answer by continent
Europe 30.00%
Asia 23.08%
Unknown 22.31%
Americas 16.15%
Oceania 4.62%
Africa 3.85%
If the tools, platforms or databases from the previous question could be deployed in Galaxy would use prefer to use in there?
Yes 77.69%
No 11.54%
Unknown 10.77%
10
25
50
75
100
Number of Responses
Proportion
20%
40%
60%
80%
