# Supplementary material for: The Microbiology Galaxy Lab: A community-driven gateway to tools, workflows, and training for reproducible and FAIR analysis of microbial data
Source: bioRxiv. 2025 Mar 26:2024.12.23.629682. Originally published 2024 Dec 27. Preprint. [Version 2] doi: 10.1101/2024.12.23.629682 (PMC11703195; doi:10.1101/2024.12.23.629682)
Supplement: Supplementary file 5 [file media-5.pdf]

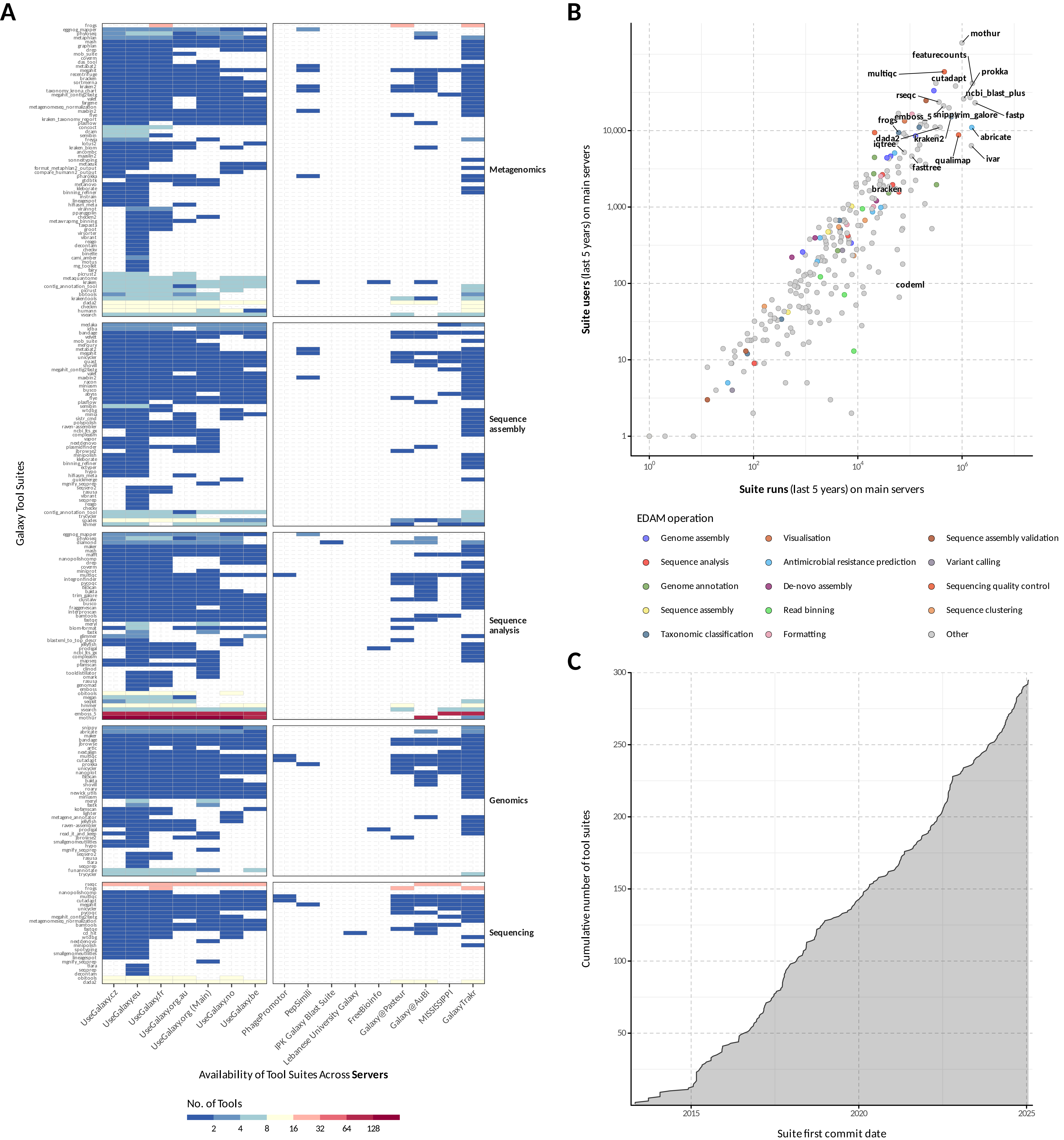

A
Metagenomics
Sequence assembly
Sequence analysis
Genomics
Sequencing
Galaxy Tool Suites
UseGalaxy.cz
UseGalaxy.eu
UseGalaxy.fr
UseGalaxy.org.au
UseGalaxy.org (Main)
UseGalaxy.no
UseGalaxy.be
PhagePromotor
PepSimili
IPK Galaxy Blast Suite
Lebanese University Galaxy
FreeBioinfo
Galaxy@Pasteur
Galaxy@AuBi
MISSISSIPPI
GalaxyTrakr
Availability of Tool Suites Across Servers
No. of Tools
2
4
8
16
32
64
128
B
mothur
featurecounts
multiqc
cutadapt
prokka
rseqc
ncbi_blast_plus
frogs
emboss_5
snippy
trim_galore
fastp
dada2
kraken2
abricate
iqtree
qualimap
ivar
fasttree
bracken
codeml
Suite users (last 5 years) on main servers
Suite runs (last 5 years) on main servers
EDAM operation
Genome assembly
Visualisation
Sequence assembly validation
Sequence analysis
Antimicrobial resistance prediction
Variant calling
Genome annotation
De-novo assembly
Sequencing quality control
Sequence assembly
Read binning
Sequence clustering
Taxonomic classification
Formatting
Other
C
Cumulative number of tool suites
Suite first commit date
2015
2020
2025
