# Supplementary material for: The Microbiology Galaxy Lab: A community-driven gateway to tools, workflows, and training for reproducible and FAIR analysis of microbial data
Source: bioRxiv. 2025 Mar 26:2024.12.23.629682. Originally published 2024 Dec 27. Preprint. [Version 2] doi: 10.1101/2024.12.23.629682 (PMC11703195; doi:10.1101/2024.12.23.629682)
Supplement: Supplementary file 6 [file media-6.pdf]

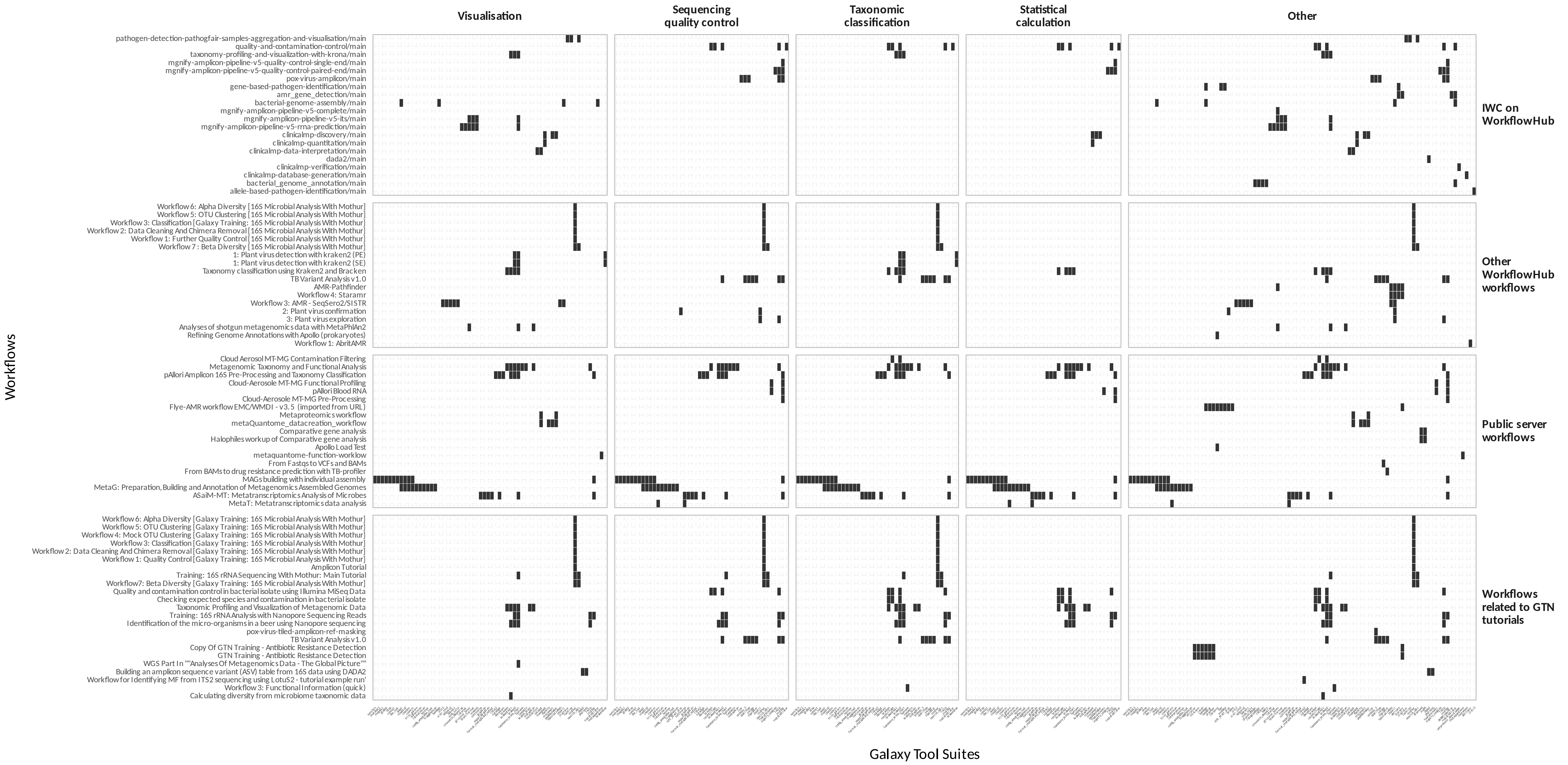

Visualisation
Sequencing quality control
Taxonomic classification
Statistical calculation
Other
IWC on WorkflowHub
Other WorkflowHub workflows
Public server workflows
Workflows related to GTN tutorials
Workflows
Galaxy Tool Suites
pathogen-detection-pathogfair-samples-aggregation-and-visualisation/main
quality-and-contamination-control/main
taxonomy-profiling-and-visualization-with-krona/main
mgnify-amplicon-pipeline-v5-quality-control-single-end/main
mgnify-amplicon-pipeline-v5-quality-control-paired-end/main
pox-virus-amplicon/main
gene-based-pathogen-identification/main
amr_gene_detection/main
bacterial-genome-assembly/main
mgnify-amplicon-pipeline-v5-complete/main
mgnify-amplicon-pipeline-v5-its/main
mgnify-amplicon-pipeline-v5-rrna-prediction/main
clinicalmp-discovery/main
clinicalmp-quantitation/main
clinicalmp-data-interpretation/main
dada2/main
clinicalmp-verification/main
clinicalmp-database-generation/main
bacterial_genome_annotation/main
allele-based-pathogen-identification/main
Workflow 6: Alpha Diversity [16S Microbial Analysis With Mothur]
Workflow 5: OTU Clustering [16S Microbial Analysis With Mothur]
Workflow 3: Classification [Galaxy Training: 16S Microbial Analysis With Mothur]
Workflow 2: Data Cleaning And Chimera Removal [16S Microbial Analysis With Mothur]
Workflow 1: Further Quality Control [16S Microbial Analysis With Mothur]
Workflow 7 : Beta Diversity [16S Microbial Analysis With Mothur]
1: Plant virus detection with kraken2 (PE)
1: Plant virus detection with kraken2 (SE)
Taxonomy classification using Kraken2 and Bracken
TB Variant Analysis v1.0
AMR-Pathfinder
Workflow 4: Staramr
Workflow 3: AMR - SeqSero2/SISTR
2: Plant virus confirmation
3: Plant virus exploration
Analyses of shotgun metagenomics data with MetaPhlAn2
Refining Genome Annotations with Apollo (prokaryotes)
Workflow 1: AbritAMR
Cloud Aerosol MT-MG Contamination Filtering
Metagenomic Taxonomy and Functional Analysis
pAllori Amplicon 16S Pre-Processing and Taxonomy Classification
Cloud-Aerosole MT-MG Functional Profiling
pAllori Blood RNA
Cloud-Aerosole MT-MG Pre-Processing
Flye-AMR workflow EMC/WMDI - v3.5 (imported from URL)
Metaproteomics workflow
metaQuantome_datacreation_workflow
Comparative gene analysis
Halophiles workup of Comparative gene analysis
Apollo Load Test
metaquantome-function-worklow
From Fastqs to VCFs and BAMs
From BAMs to drug resistance prediction with TB-profiler
MAGs building with individual assembly
MetaG: Preparation, Building and Annotation of Metagenomics Assembled Genomes
ASaiM-MT: Metatranscriptomics Analysis of Microbes
MetaT: Metatranscriptomics data analysis
Workflow 6: Alpha Diversity [Galaxy Training: 16S Microbial Analysis With Mothur]
Workflow 5: OTU Clustering [Galaxy Training: 16S Microbial Analysis With Mothur]
Workflow 4: Mock OTU Clustering [Galaxy Training: 16S Microbial Analysis With Mothur]
Workflow 3: Classification [Galaxy Training: 16S Microbial Analysis With Mothur]
Workflow 2: Data Cleaning And Chimera Removal [Galaxy Training: 16S Microbial Analysis With Mothur]
Workflow 1: Quality Control [Galaxy Training: 16S Microbial Analysis With Mothur]
Amplicon Tutorial
Training: 16S rRNA Sequencing With Mothur: Main Tutorial
Workflow7: Beta Diversity [Galaxy Training: 16S Microbial Analysis With Mothur]
Quality and contamination control in bacterial isolate using Illumina MiSeq Data
Checking expected species and contamination in bacterial isolate
Taxonomic Profiling and Visualization of Metagenomic Data
Training: 16S rRNA Analysis with Nanopore Sequencing Reads
Identification of the micro-organisms in a beer using Nanopore sequencing
pox-virus-tiled-amplicon-ref-masking
TB Variant Analysis v1.0
Copy Of GTN Training - Antibiotic Resistance Detection
GTN Training - Antibiotic Resistance Detection
WGS Part In ""Analyses Of Metagenomics Data - The Global Picture""
Building an amplicon sequence variant (ASV) table from 16S data using DADA2
Workflow for Identifying MF from ITS2 sequencing using LotuS2 - tutorial example run'
Workflow 3: Functional Information (quick)
Calculating diversity from microbiome taxonomic data
