# Supplementary material for: The Microbiology Galaxy Lab: A community-driven gateway to tools, workflows, and training for reproducible and FAIR analysis of microbial data
Source: bioRxiv. 2025 Mar 26:2024.12.23.629682. Originally published 2024 Dec 27. Preprint. [Version 2] doi: 10.1101/2024.12.23.629682 (PMC11703195; doi:10.1101/2024.12.23.629682)
Supplement: Supplementary file 7 [file media-7.pdf]

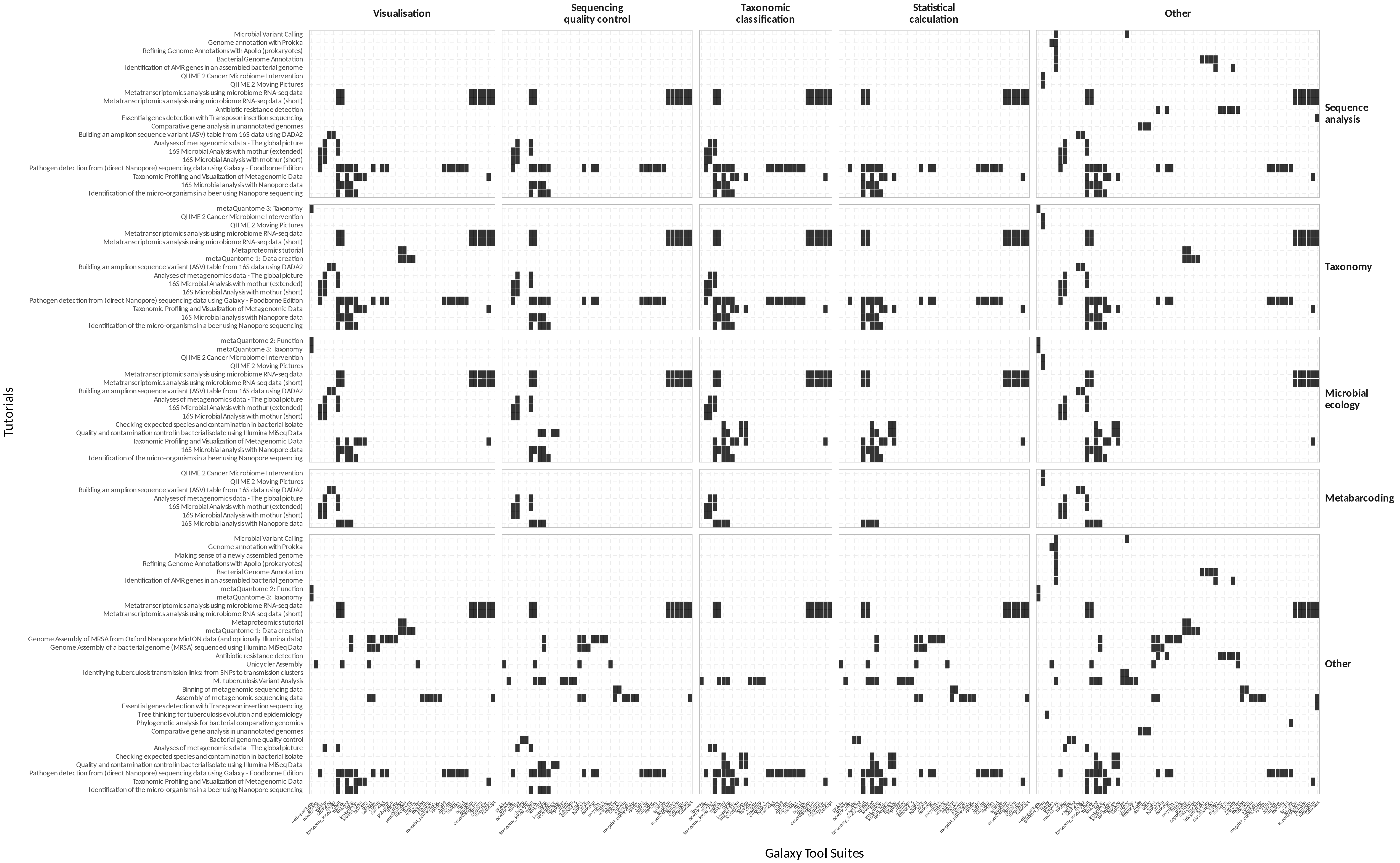

Visualisation
Sequencing quality control
Taxonomic classification
Statistical calculation
Other
Microbial Variant Calling
Genome annotation with Prokka
Refining Genome Annotations with Apollo (prokaryotes)
Bacterial Genome Annotation
Identification of AMR genes in an assembled bacterial genome
QIIME 2 Cancer Microbiome Intervention
QIIME 2 Moving Pictures
Metatranscriptomics analysis using microbiome RNA-seq data
Metatranscriptomics analysis using microbiome RNA-seq data (short)
Antibiotic resistance detection
Essential genes detection with Transposon insertion sequencing
Comparative gene analysis in unannotated genomes
Building an amplicon sequence variant (ASV) table from 16S data using DADA2
Analyses of metagenomics data - The global picture
16S Microbial Analysis with mothur (extended)
16S Microbial Analysis with mothur (short)
Pathogen detection from (direct Nanopore) sequencing data using Galaxy - Foodborne Edition
Taxonomic Profiling and Visualization of Metagenomic Data
16S Microbial analysis with Nanopore data
Identification of the micro-organisms in a beer using Nanopore sequencing
Sequence analysis
metaQuantome 3: Taxonomy
QIIME 2 Cancer Microbiome Intervention
QIIME 2 Moving Pictures
Metatranscriptomics analysis using microbiome RNA-seq data
Metatranscriptomics analysis using microbiome RNA-seq data (short)
Metaproteomics tutorial
metaQuantome 1: Data creation
Building an amplicon sequence variant (ASV) table from 16S data using DADA2
Analyses of metagenomics data - The global picture
16S Microbial Analysis with mothur (extended)
16S Microbial Analysis with mothur (short)
Pathogen detection from (direct Nanopore) sequencing data using Galaxy - Foodborne Edition
Taxonomic Profiling and Visualization of Metagenomic Data
16S Microbial analysis with Nanopore data
Identification of the micro-organisms in a beer using Nanopore sequencing
Taxonomy
metaQuantome 2: Function
metaQuantome 3: Taxonomy
QIIME 2 Cancer Microbiome Intervention
QIIME 2 Moving Pictures
Metatranscriptomics analysis using microbiome RNA-seq data
Metatranscriptomics analysis using microbiome RNA-seq data (short)
Building an amplicon sequence variant (ASV) table from 16S data using DADA2
Analyses of metagenomics data - The global picture
16S Microbial Analysis with mothur (extended)
16S Microbial Analysis with mothur (short)
Checking expected species and contamination in bacterial isolate
Quality and contamination control in bacterial isolate using Illumina MiSeq Data
Taxonomic Profiling and Visualization of Metagenomic Data
16S Microbial analysis with Nanopore data
Identification of the micro-organisms in a beer using Nanopore sequencing
Microbial ecology
QIIME 2 Cancer Microbiome Intervention
QIIME 2 Moving Pictures
Building an amplicon sequence variant (ASV) table from 16S data using DADA2
Analyses of metagenomics data - The global picture
16S Microbial Analysis with mothur (extended)
16S Microbial Analysis with mothur (short)
16S Microbial analysis with Nanopore data
Metabarcoding
Microbial Variant Calling
Genome annotation with Prokka
Making sense of a newly assembled genome
Refining Genome Annotations with Apollo (prokaryotes)
Bacterial Genome Annotation
Identification of AMR genes in an assembled bacterial genome
metaQuantome 2: Function
metaQuantome 3: Taxonomy
Metatranscriptomics analysis using microbiome RNA-seq data
Metatranscriptomics analysis using microbiome RNA-seq data (short)
Metaproteomics tutorial
metaQuantome 1: Data creation
Genome Assembly of MRSA from Oxford Nanopore MinION data (and optionally Illumina data)
Genome Assembly of a bacterial genome (MRSA) sequenced using Illumina MiSeq Data
Antibiotic resistance detection
Unicycler Assembly
Identifying tuberculosis transmission links: from SNPs to transmission clusters
M. tuberculosis Variant Analysis
Binning of metagenomic sequencing data
Assembly of metagenomic sequencing data
Essential genes detection with Transposon insertion sequencing
Tree thinking for tuberculosis evolution and epidemiology
Phylogenetic analysis for bacterial comparative genomics
Comparative gene analysis in unannotated genomes
Bacterial genome quality control
Analyses of metagenomics data - The global picture
Checking expected species and contamination in bacterial isolate
Quality and contamination control in bacterial isolate using Illumina MiSeq Data
Pathogen detection from (direct Nanopore) sequencing data using Galaxy - Foodborne Edition
Taxonomic Profiling and Visualization of Metagenomic Data
Identification of the micro-organisms in a beer using Nanopore sequencing
Other
Tutorials
Galaxy Tool Suites
