# Supplementary material for: microGalaxy: A gateway to tools, workflows, and training for reproducible and FAIR analysis of microbial data
Source: bioRxiv. 2024 Dec 27:2024.12.23.629682. Preprint. [Version 1] doi: 10.1101/2024.12.23.629682 (PMC11703195; doi:10.1101/2024.12.23.629682)
Supplement: Supplementary file 1 [file NIHPP2024.12.23.629682V1-supplement-1.pdf]

bioRxiv preprint doi: https://doi.org/10.1101/2024.12.23.629682; this version posted December 27, 2024. The copyright holder for this preprint (which was not certified by peer review) is the author/funder, who has granted bioRxiv a license to display the preprint in perpetuity. It is made available under aCC-BY 4.0 International license.

Supplementary information

Supplementary Figures

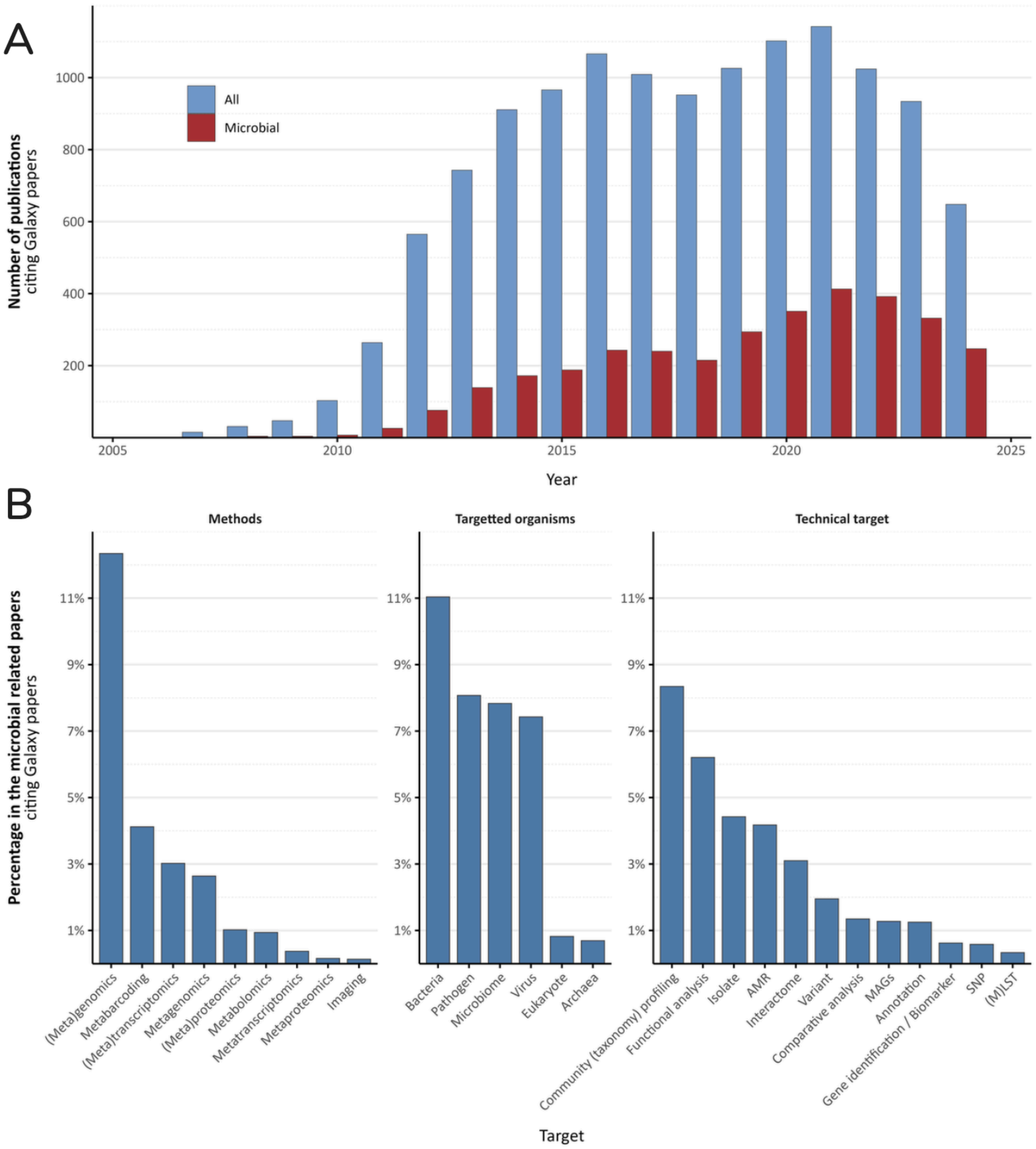

Supplementary Figure 1. Citation Trends and Microbial Research Topics in Galaxy Publications. The citations were extracted from the Galaxy Project's Google Scholar profile, with additional details retrieved using Semantic Search. (A) Annual publication trends show the total number of citations (blue) alongside those specific to microbial research (brown) (B) Breakdown of microbial-focused citations by Targeted Organisms, Technical Targets, and Methods. Categories were annotated based on predefined keywords found in the title or abstract of each publication

bioRxiv preprint doi: https://doi.org/10.1101/2024.12.23.629682; this version posted December 27, 2024. The copyright holder for this preprint (which was not certified by peer review) is the author/funder, who has granted bioRxiv a license to display the preprint in perpetuity. It is made available under aCC-BY 4.0 International license.

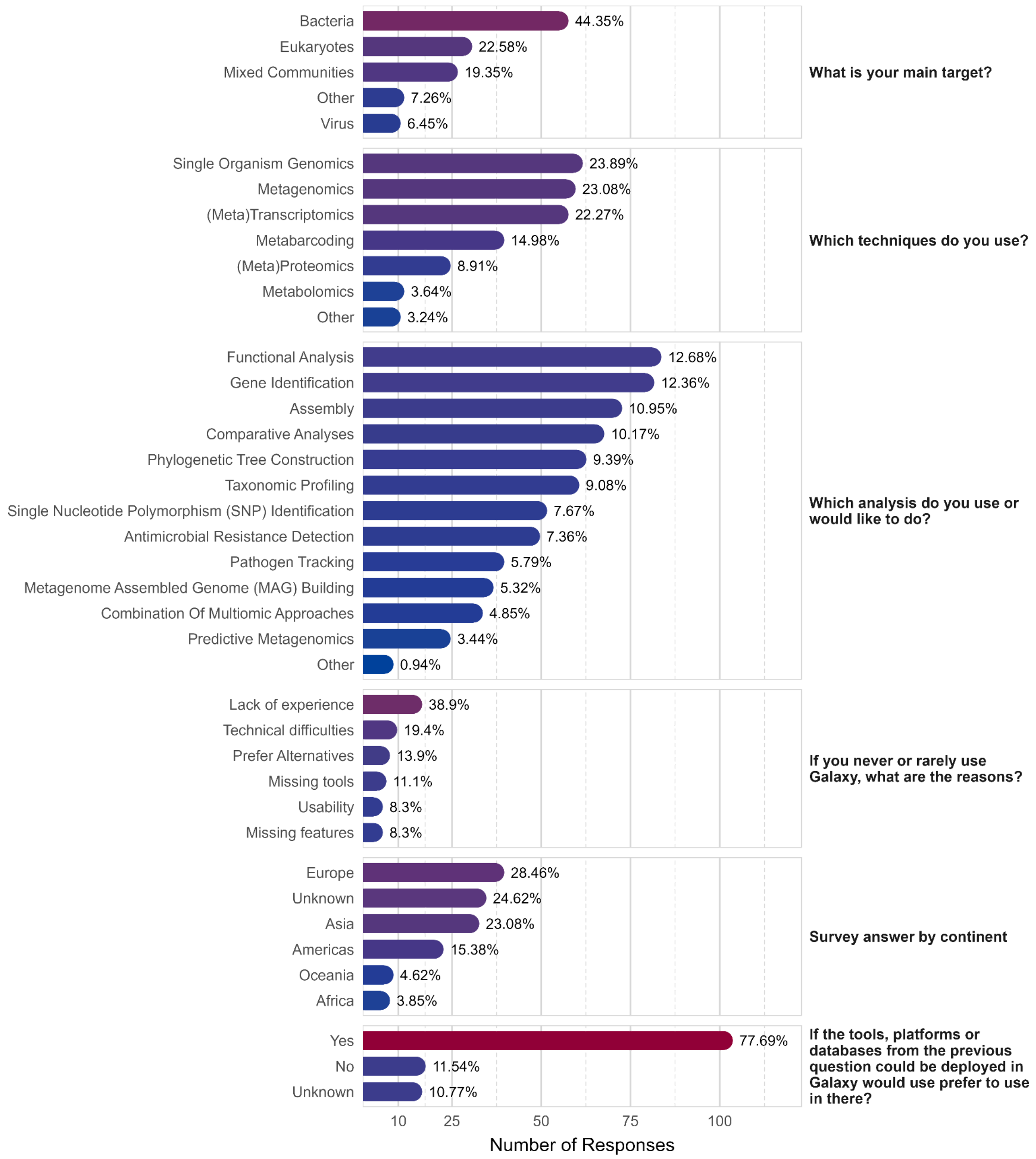

Supplementary Figure 2. Survey results from the microbial research community (March–September 2023). Anonymous responses highlighting the main research targets, techniques, and analyses used or desired by microbial researchers. The figure also reports challenges faced by users who rarely or never use Galaxy, survey responses by continent, and preferences regarding tool deployment in Galaxy.

bioRxiv preprint doi: https://doi.org/10.1101/2024.12.23.629682; this version posted December 27, 2024. The copyright holder for this preprint (which was not certified by peer review) is the author/funder, who has granted bioRxiv a license to display the preprint in perpetuity. It is made available under aCC-BY 4.0 International license.

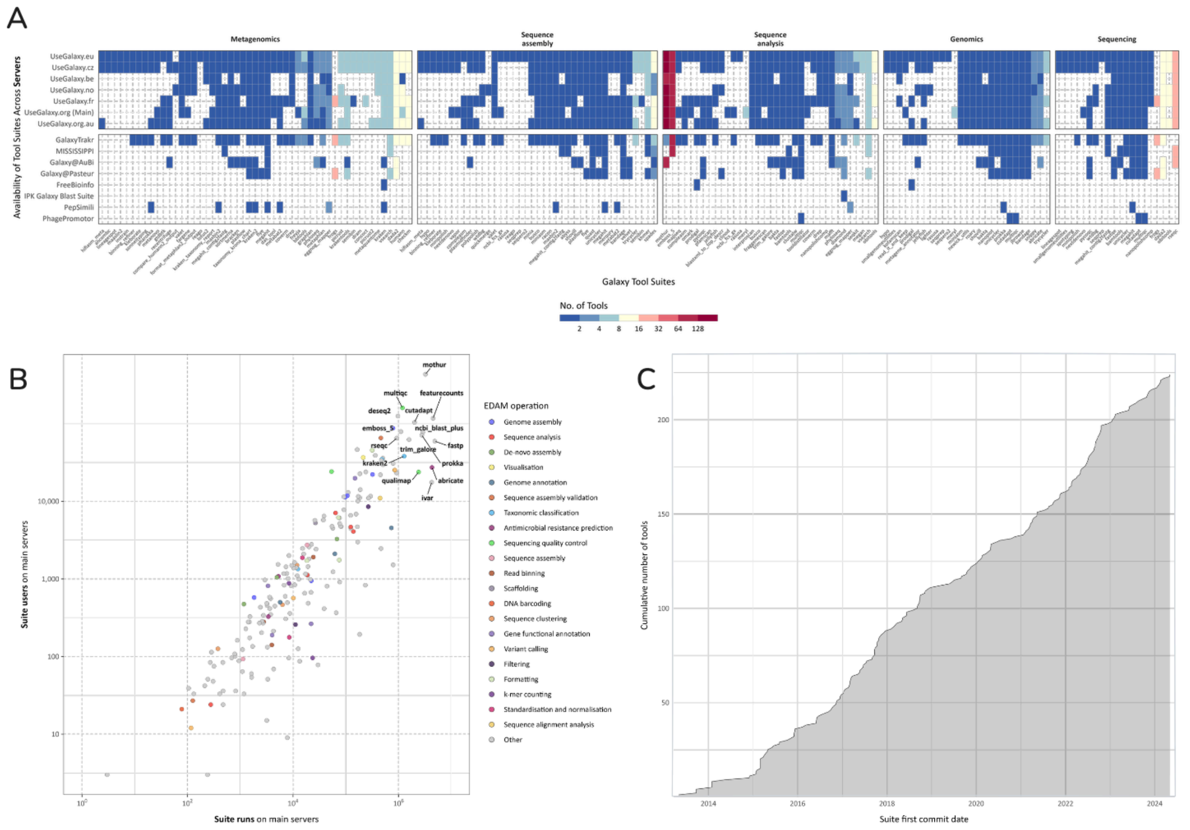

Supplementary Figure 3. Availability, usability, and growth of microGalaxy tool suites within the Galaxy ecosystem. (A) Heatmap illustrating the availability of microGalaxy tool suites (x-axis) across various Galaxy servers (y-axis), grouped by EDAM topics. Tool suites may appear in multiple clusters, as they can be annotated with several topics. A logarithmic scale is applied for improved visualization. (B) Scatter plots showing the usability of microGalaxy tool suites over the past five years across all Galaxy main servers, categorized by their EDAM operations. The x-axis represents the total number of tool users, while the y-axis also denotes the total number of tool users (potentially correct this redundancy). (C) Cumulative number of tools added to Galaxy over time, based on the date of the first commit for each tool suite.

bioRxiv preprint doi: https://doi.org/10.1101/2024.12.23.629682; this version posted December 27, 2024. The copyright holder for this preprint (which was not certified by peer review) is the author/funder, who has granted bioRxiv a license to display the preprint in perpetuity. It is made available under aCC-BY 4.0 International license.

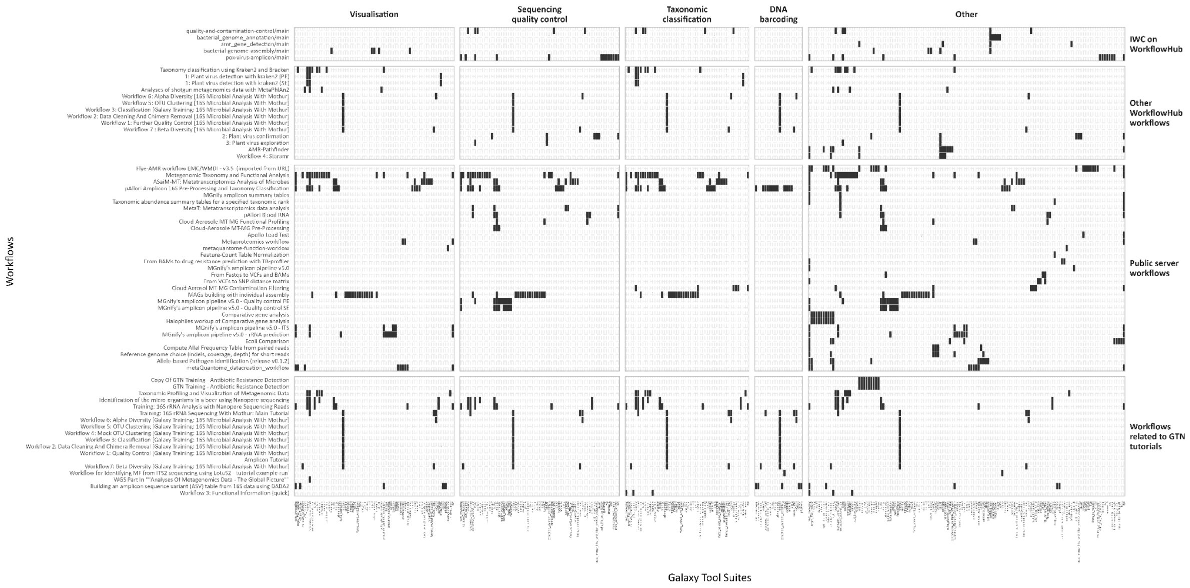

Supplementary Figure 4. Usage of microGalaxy tool suites across workflows. Heatmap illustrating the presence of microGalaxy tool suites within the available microGalaxy workflows, grouped by three levels of development. The tool suites are organized based on their corresponding EDAM operations, highlighting the breadth of tools utilized for different workflows.

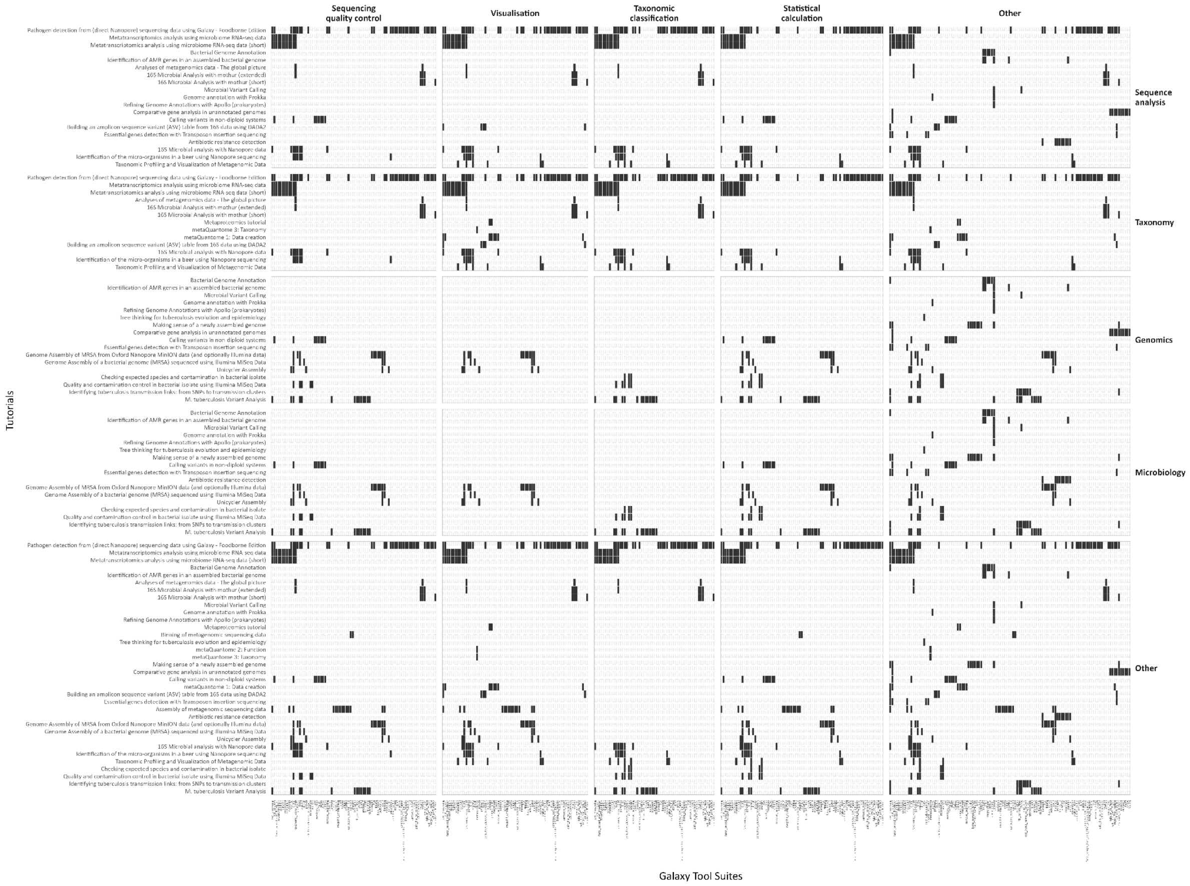

bioRxiv preprint doi: https://doi.org/10.1101/2024.12.23.629682; this version posted December 27, 2024. The copyright holder for this preprint (which was not certified by peer review) is the author/funder, who has granted bioRxiv a license to display the preprint in perpetuity. It is made available under aCC-BY 4.0 International license.

Supplementary Figure 5. Usage of microGalaxy tool suites across training materials. Heatmap illustrating the presence of microGalaxy tool suites within the available microGalaxy training materials, grouped by EDAM topics. The tool suites are organized based on their corresponding EDAM operations, highlighting the breadth of tools utilized for different training contexts.

Supplementary Tables

Supplementary Table 1. Features and capabilities of 44 existing user-friendly microbial data analysis platforms (non-exhaustive list). The table includes columns detailing general characteristics (e.g., free usage, open source, workflow/pipeline manager), accessibility and availability (e.g., simple end-user modification, user-friendly interface, publicly available web server, automatable API, last update), user support and documentation (e.g., tutorials, documentation, user support), possible methods used to generate input data (e.g., isolate genomics, amplicon/metabarcoding, WGS/metagenomics, metatranscriptomics), and supported analyses (e.g., QC, taxonomy profiling, MAGs, comparative omics, multi-omics integration, interactive visualizations).

Supplementary Table 2. **Anonymous results from the microbial research community survey conducted between March and September 2023.** The table includes aggregated and anonymised responses to survey questions, detailing researchers' demographics, tool usage, training needs, and suggestions for future improvements to the Galaxy platform.

Supplementary Table 3. **Overview of the 224 tools available in the microGalaxy tool set.** The table includes detailed metadata for each tool suite, such as suite ID, tool IDs, description, first commit date, homepage, version, conda package details, version status, and ToolShed categories. It also incorporates EDAM annotations (operations, reduced operations, topics, reduced topics) and links to external resources like bio.tools (ID, name, description). Usage statistics from Galaxy's main servers over the last five years, including the number of users and tool runs, are also provided to highlight adoption and activity levels.

Supplementary Table 4. **Comprehensive list of microGalaxy workflows**. This table documents 69 workflows, detailing their names, sources, unique identifiers, and links to relevant repositories. Additional information includes the creators, associated tags, creation and update timestamps, latest and available versions, number of steps, and tools used. The workflows are categorized by EDAM operations and topics, and information on licensing, DOI assignments, and related projects is provided.

Supplementary Table 5. **Overview of microGalaxy-related training events conducted between 2019 and 2024.** The table provides details for 38 events, including their start and end dates, titles, covered topics, target audiences, and the specific contributions of microGalaxy when the event encompassed broader subjects beyond microbial data analysis. Links to event pages and the resources utilized during the training sessions are also included.

Supplementary Table 6. Summary of use case submissions collected from survey participants. The table includes anonymised details of each use case, such as the research question, experimental methods, data analysis approaches, Galaxy tools and workflows used, and specific benefits of using Galaxy. It also highlights the status of the use cases

bioRxiv preprint doi: https://doi.org/10.1101/2024.12.23.629682; this version posted December 27, 2024. The copyright holder for this preprint (which was not certified by peer review) is the author/funder, who has granted bioRxiv a license to display the preprint in perpetuity. It is made available under aCC-BY 4.0 International license.

(e.g., starting, ongoing, completed) and any associated publications. This summary serves as a foundation for the Discussion section of the manuscript.

Supplementary Documents

Supplementary Document 1. **Complete list of survey questions shared with the microbial research community via a Google Form between March and September 2023**. The survey aimed to assess researchers' needs, challenges, and preferences using microbial data analysis tools and platforms.

Supplementary Document 2. **Template to collect detailed use cases from survey participants who expressed interest in contributing to the planned manuscript**. The document includes sections for contact details, research questions, methods, tools, workflows, and results, along with prompts for providing a detailed description of the use cases. Preliminary survey results are also shared to provide context and highlight the motivation for the study.
